# The VCAM1–ApoE pathway directs microglial chemotaxis and alleviates Alzheimer’s disease pathology

**DOI:** 10.1038/s43587-023-00491-1

**Published:** 2023-09-21

**Authors:** Shun-Fat Lau, Wei Wu, Hiu Yi Wong, Li Ouyang, Yi Qiao, Jiahui Xu, Jessica Hiu-Yan Lau, Carlton Wong, Yuanbing Jiang, David M. Holtzman, Amy K. Y. Fu, Nancy Y. Ip

**Affiliations:** 1 Division of Life Science, State Key Laboratory of Molecular Neuroscience, Molecular Neuroscience Center, The Hong Kong University of Science and Technology, Clear Water Bay, Hong Kong, China; 2 Hong Kong Center for Neurodegenerative Diseases, Hong Kong Science Park, Hong Kong, China; 3 Department of Neurology, Hope Center for Neurological Disorders, Charles F. and Joanne Knight Alzheimer’s Disease Research Center, Washington University School of Medicine, St. Louis, MO USA; 4 Guangdong Provincial Key Laboratory of Brain Science, Disease and Drug Development, HKUST Shenzhen Research Institute, Shenzhen–Hong Kong Institute of Brain Science, Shenzhen, Guangdong China

**Keywords:** Alzheimer's disease, Cellular neuroscience, Ageing

## Abstract

In Alzheimer’s disease (AD), sensome receptor dysfunction impairs microglial danger-associated molecular pattern (DAMP) clearance and exacerbates disease pathology. Although extrinsic signals, including interleukin-33 (IL-33), can restore microglial DAMP clearance, it remains largely unclear how the sensome receptor is regulated and interacts with DAMP during phagocytic clearance. Here, we show that IL-33 induces VCAM1 in microglia, which promotes microglial chemotaxis toward amyloid-beta (Aβ) plaque-associated ApoE, and leads to Aβ clearance. We show that IL-33 stimulates a chemotactic state in microglia, characterized by Aβ-directed migration. Functional screening identified that VCAM1 directs microglial Aβ chemotaxis by sensing Aβ plaque-associated ApoE. Moreover, we found that disrupting VCAM1–ApoE interaction abolishes microglial Aβ chemotaxis, resulting in decreased microglial clearance of Aβ. In patients with AD, higher cerebrospinal fluid levels of soluble VCAM1 were correlated with impaired microglial Aβ chemotaxis. Together, our findings demonstrate that promoting VCAM1–ApoE-dependent microglial functions ameliorates AD pathology.

## Abstract (web summary)

A single-cell transcriptomic analysis by Lau et al. reveals a homeostatic–chemotactic–phagocytic state transition in microglia upon IL-33 stimulation, identifying VCAM1 as a key regulator of microglial chemotaxis by sensing Aβ plaque-associated ApoE.

## Main

In Alzheimer’s disease (AD), microglial clearance regulates the turnover of neurotoxic danger-associated molecular patterns (DAMPs), including amyloid-beta (Aβ), hyperphosphorylated tau and dystrophic neurites^1–5^. Microglial DAMP clearance is controlled by a stepwise functional transition in which microglia first migrate toward DAMPs and subsequently perform phagocytic clearance^3,6,7^. To sense and interact with DAMPs, microglia express a repertoire of surface sensome receptors that specifically bind to their cognate ligands on DAMPs and trigger microglial activation^8–12^. Therefore, sensome receptor–DAMP interactions are critical for the microglial clearance of DAMPs, which in turn limits AD pathogenesis.

During DAMP clearance, microglia modify their sensome receptor expression profile while transitioning between functional states. In response to Aβ aggregation, microglia adopt a phagocytic phenotype and express the gene signature of disease-associated microglia (DAM) (also referred to as neurodegenerative microglia or activated-response microglia) marked by increased expression of sensome receptors (that is, *Axl* and *Trem2*)^13–15^. Genetic analysis further shows that these sensome receptors are important for regulating AD pathogenesis. In particular, many AD risk variants and mutations are located near or in the coding sequences of genes that encode sensome receptors, including *CD33* and *TREM2*^16–18^. These variants or mutations reduce the microglial phagocytic clearance, barrier formation around DAMPs, and exacerbate AD pathogenesis by altering the expression, function or cleavage of their respective sensome receptors^10,19–21^. Interestingly, some sensome receptors, such as TREM2, can regulate microglial functions and AD pathology in a stage-dependent manner^22^. Therefore, further investigations are required to understand how these sensome receptors and their dysfunctioning regulate microglial functions and contribute to disease pathogenesis in AD.

It remains largely unknown how sensome receptors are regulated and interact with DAMPs to control specific functions during microglial DAMP clearance, including chemotaxis and phagocytosis. The induction of microglial DAMP clearance is temporally stochastic in vivo, partly because Aβ deposits develop in a spatiotemporally random manner. This hinders detailed investigations of the different functional states of microglia and their regulatory mechanisms during DAMP clearance. Nonetheless, our previous study demonstrates that interleukin-33 (IL-33) promotes microglial Aβ clearance in a temporally precise manner. By performing two-photon in vivo imaging and flow cytometry analyses, we showed that microglia first exhibit Aβ chemotaxis (3–12 h after injection) and subsequently phagocytose Aβ (15–24 h after injection) in APP/PS1 mice, a mouse model of amyloidosis^23^. Therefore, in the present study, we investigated how sensome receptors and their interactions with their cognate ligands regulate microglial DAMP clearance upon IL-33 treatment in APP/PS1 mice. Addressing these knowledge gaps provides important insights into the role of sensome receptor–ligand interactions in microglial DAMP clearance in AD.

Here, we demonstrate that VCAM1 induction in microglia enhances their interaction with the cognate ligand ApoE to drive Aβ chemotaxis and subsequent Aβ clearance. Single-cell transcriptomic and lineage development analyses revealed that in IL-33-treated APP/PS1 mice, microglia adopt a chemotactic state in which they exhibit Aβ-directed migration before transitioning into a Aβ phagocytic state. Moreover, functional screening identified that VCAM1 regulates microglial chemotaxis toward Aβ plaques through sensing ApoE in Aβ plaques. Blockade of VCAM1–ApoE interaction inhibits the Aβ chemotaxis of microglia and their subsequent differentiation into phagocytic microglia after IL-33 treatment. In addition, in the brains of patients with AD, VCAM1 signaling is inhibited and correlated with impaired microglial migration toward Aβ plaques. Together, our findings demonstrate a VCAM1–ApoE pathway that is important for promoting microglial chemotaxis toward Aβ plaques and alleviating amyloid pathology.

## Results

### IL-33RM undergo a stepwise functional state transition

**Fig. 1:**
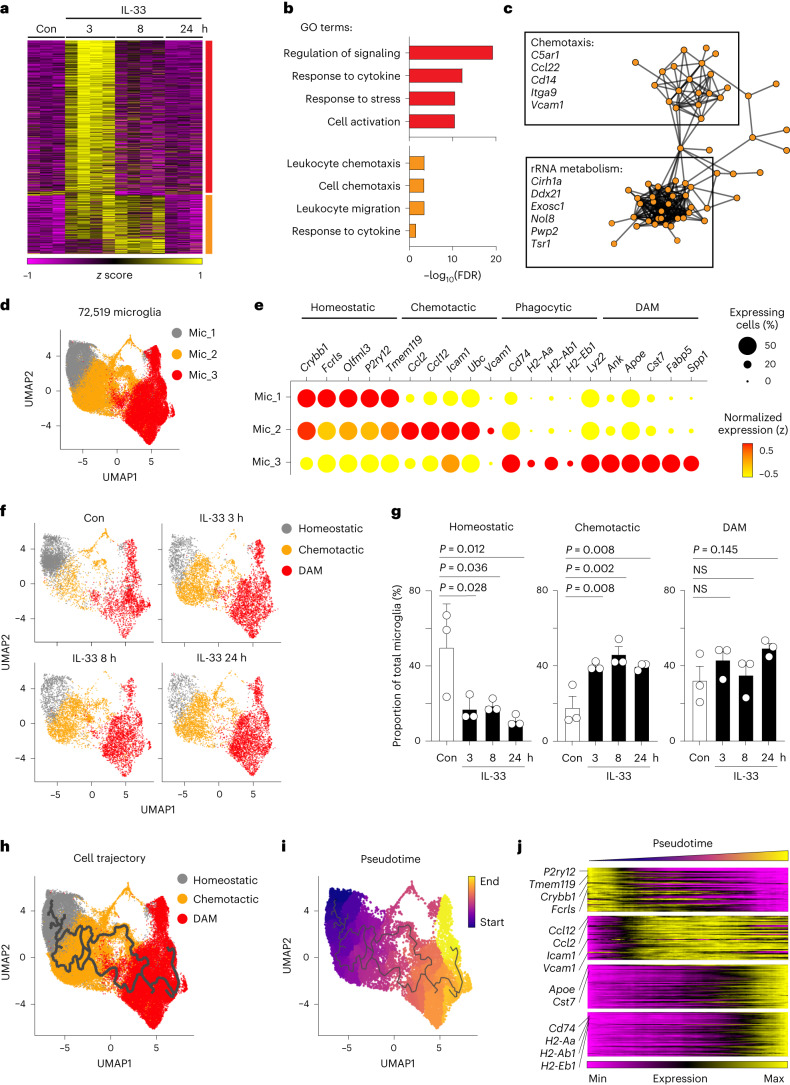
IL-33-responsive microglia undergo stepwise transcriptomic reprogramming. **a**–**c**, IL-33 induces prolonged expression of the microglial chemotactic gene signature. **a**, Heatmap showing the expression levels of 1,433 IL-33-induced genes in microglia 3, 8 and 24 h after IL-33 treatment (adjusted *P* value < 0.05). The bar on the far right indicates genes showing transient activation (red) and prolonged activation (orange). **b**, Bar plot showing the top GO pathways associated with the genes showing transient (red) and prolonged (orange) activation (as in panel a). FDR, false discovery rate. **c**, Protein–protein interaction analysis of genes exhibiting prolonged activation. **d**–**g**, IL-33 regulates microglial heterogeneity in a sequential manner. **d**, Uniform Manifold Approximation and Projection (UMAP) plot of three microglial subtypes showing unbiased clustering of 72,519 microglia from APP/PS1 mice treated with IL-33 or control for 3, 8, or 24 h (each condition corresponds to 3 biological independent samples). **e**, Dot plot showing the expression levels of the top signature genes of the 3 microglial subtypes. **f**,**g**, UMAP plots (f) and bar plots (g) showing the proportions of homeostatic, chemotactic and DAM in the four conditions (*n* = 3 mice in each condition; one-way ANOVA with Dunnett’s multiple comparisons test). Con, control. **h–j**, The developmental lineage of IL-33RM involves the sequential homeostatic–chemotactic–phagocytic state transition. UMAP plots showing the cell trajectory (h) and pseudotime ordering (i) of the IL-33RM developmental lineage. **j**, Heatmap visualizing the smoothed expression levels of the top homeostatic, chemotactic, DAM, and phagocytic signature genes (as described in the text) along the IL-33RM developmental lineage. All data are mean ± standard error of the mean (s.e.m.). Source data

During IL-33-stimulated Aβ clearance, microglia first undergo Aβ chemotaxis and subsequently adopt an MHC-II^+^ phagocytic phenotype; microglia of this DAM subtype are referred to as IL-33-responsive microglia (IL-33RM)^23^. However, the molecular mechanism by which how microglia sequentially adopt chemotactic and phagocytic phenotypes during microglial Aβ clearance is poorly understood. Therefore, we first profiled the transcriptomes of microglia in APP/PS1 mice in the chemotactic state (that is, 3 and 8 h after IL-33 treatment) and the phagocytic state (that is, 24 h after IL-33 treatment). Bulk RNA sequencing (RNA-seq) analysis showed that the expression levels of 1,433 genes were elevated in microglia when they started to migrate toward Aβ plaques (that is, 3 h after IL-33 treatment); the expressions of 381 genes remained elevated throughout the chemotactic state (that is, until 8 h after IL-33 treatment) (Fig. 1a). Gene Ontology (GO) and protein–protein interaction network analyses showed that these 381 activated genes are functionally associated with cell chemotaxis and migration (Fig. 1b) and form two major gene hubs that are associated with chemotaxis (for example, *C5ar1*, *Cd14* and *Vcam1*) and rRNA metabolism (for example, *Cirh1a*, *Exosc1* and *Tsr1*) (Fig. 1c). These findings show that after IL-33 treatment, microglia express a chemotactic gene signature before adopting an Aβ phagocytic phenotype.

**Extended Data Fig. 1:**
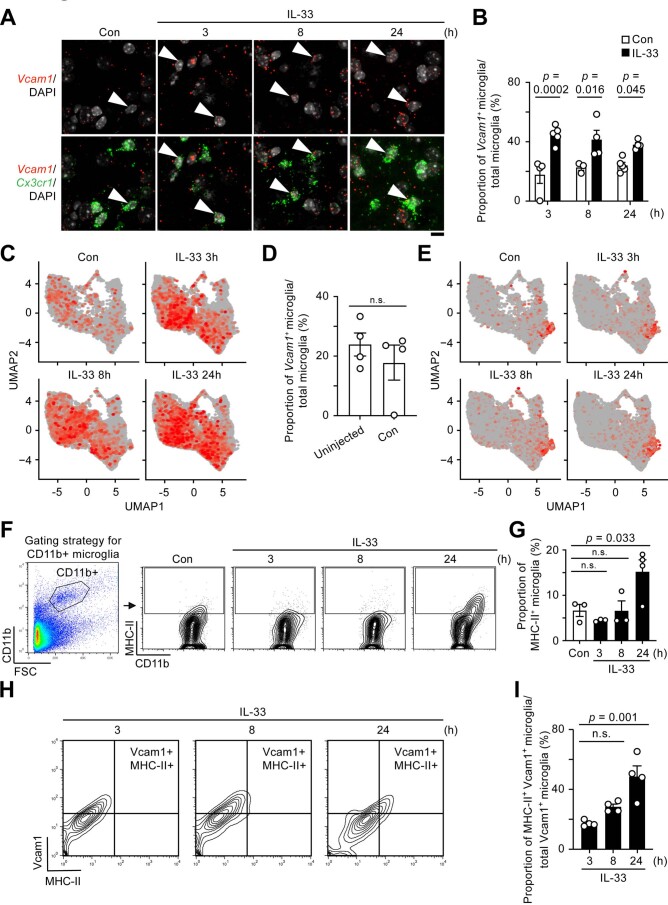
Chemotactic microglia migrate toward amyloid-beta plaques after interleukin-33 treatment. **a-c**, The induction of chemotactic microglia lasts throughout the chemotactic phase. **a**,**b**, Representative images (a) and quantification (b) showing the proportion of chemotactic microglia (that is, *Vcam1*^+^ *Cx3cr1*^+^ cells) after interleukin-33 (IL-33) treatment (3 h Con: *n* = 4; 3 h IL-33: *n* = 5; 8 h Con: *n* = 3; 8 h IL-33: *n* = 4; 24 h Con: *n* = 5; 24 h IL-33: *n* = 4; two-way ANOVA with Šidák’s multiple comparisons test). Arrowheads indicate *Vcam1*-expressing microglia. Scale bar = 10 μm. **c**, UMAP plots showing *Vcam1* expression across conditions. **d**, Bar plot quantifying the proportions of chemotactic microglia (that is, *Vcam1*^+^ *Cx3cr1*^+^ cells) in uninjected and PBS-injected (Con) APP/PS1 mice (*n* = 4 mice per condition; two-tailed unpaired Student’s *t*-test). **e**, UMAP plots showing *H2-Ab1* expression across conditions. **f**,**g**, Induction of MHC-II^+^ phagocytic microglia after IL-33 treatment. Representative contour plots (f) and quantification (g) showing the proportions of MHC-II^+^ phagocytic microglia (that is, MHC-II^+^ CD11b^+^ cells) after IL-33 treatment (Con: *n* = 3 mice; 3 h: *n* = 3 mice; 8 h: *n* = 3 mice; 24 h: *n* = 4 mice; one-way ANOVA with Dunnett’s multiple comparisons test). **h**,**i**, Chemotactic microglia gradually express MHC-II after IL-33 treatment. Representative contour plots (h) and quantification (i) showing the proportions of MHC-II–expressing chemotactic microglia (that is, MHC-II ^+^ VCAM1 ^+^ CD11b^+^ cells) after IL-33 treatment (3 h: *n* = 4 mice; 8 h: *n* = 4 mice; 24 h: *n* = 4 mice; one-way ANOVA with Dunnett’s multiple comparisons test). All data are mean ± s.e.m. Source data

Given that microglia are highly heterogeneous, we subsequently examined whether the induced expression of the chemotactic gene signature in microglia is due to transcriptomic changes in a specific microglial subpopulation(s). Using single-cell RNA-seq (scRNA-seq) analysis, we identified 3 microglial subclusters—termed Mic_1 to Mic_3—in IL-33–treated APP/PS1 mice and determined their respective signature genes (Fig. 1d). Transcriptomic analysis showed that the Mic_1 subcluster (for example, *Fcrls*, *P2ry12*, and *Tmem119*), Mic_2 subcluster (for example, *Ccl2*, *Ccl12*, *Icam1*, and *Vcam1*), and Mic_3 subcluster (for example, *Apoe*, *Cst7*, and *Spp1*) express homeostatic, chemotactic, and DAM gene signatures, respectively (Fig. 1e)^13,14^. Moreover, the MHC-II–expressing IL-33RM clustered together with the DAM (Fig. 1e), which is concordant with our previous findings^23^. Therefore, we classified Mic_1 to Mic_3 as homeostatic microglia, chemotactic microglia and DAM, respectively. In addition, upon IL-33 treatment, the proportion of chemotactic microglia increased ~2.2-fold throughout the chemotactic phase (that is, 3–8 h after IL-33 treatment), which coincided with the reduced proportion of homeostatic microglia (Fig. 1f,g). We then conducted in situ hybridization analysis to validate the IL-33-induced changes in the proportion of chemotactic microglia in APP/PS1 mice. By monitoring *Vcam1*, a top chemotactic signature gene whose expression remains elevated for over 8 h after IL-33 treatment (Fig. 1a), we found that the proportion of chemotactic microglia (that is, *Vcam1*^+^ microglia) increased ~1.9-fold throughout the chemotactic phase after IL-33 treatment (Extended Data Fig. 1a–c). Moreover, this induction of chemotactic microglia was not caused by the technical artifacts introduced by surgical injuries (Extended Data Fig. 1d). In contrast, flow cytometry analysis showed that MHC-II^+^ phagocytic microglia were induced 24 h after IL-33 treatment but not during the earlier chemotactic phase (Extended Data Fig. 1e–g). These results suggest that after IL-33 stimulation, the induction of a chemotactic microglial subpopulation leads to elevated expression of a chemotactic gene signature in microglia before the induction of MHC-II^+^ phagocytic microglia.

Interestingly, flow cytometry analysis showed that some IL-33–induced chemotactic microglia (that is, VCAM1^+^ microglia) gradually expressed MHC-II and became VCAM1^+^ MHC-II^+^ microglia (Extended Data Fig. 1h,i). This suggests that VCAM1^+^ chemotactic microglia are the precursors of MHC-II^+^ phagocytic microglia. We further examined the lineage relationship between these 2 microglial states by performing pseudotemporal ordering analysis. Both cell trajectory and pseudotime analyses of microglia in IL-33-treated APP/PS1 mice showed that microglia transition from a homeostatic to chemotactic to DAM state (Fig. 1h,i). Visualization of the transcriptomic signatures of microglia in each state along a pseudotime axis revealed a gradual decrease in the expression of homeostatic signature genes (that is, *P2ry12* and *Tmem119*); this was followed by an induction of chemotactic signature genes (that is, *Icam1*, *Tlr2*, and *Vcam1*), an induction of DAM signature genes (that is, *Apoe* and *Cst7*), and finally an induction of phagocytic signature genes (that is, *H2-Aa*, *H2-Ab1* and *H2-Eb1*) (Fig. 1j). Taken together, these results demonstrate that the lineage development of IL-33RM involves a sequential transition from a homeostatic state to a chemotactic state to a phagocytic state.

### ST2 is required for the induction of chemotactic microglia

**Fig. 2:**
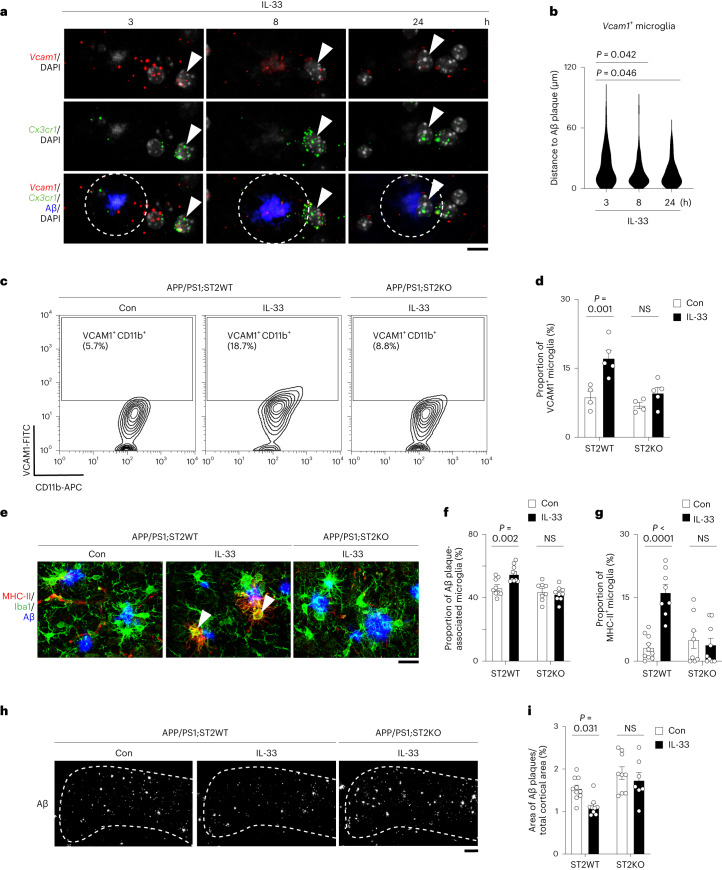
ST2-dependent induction of chemotactic microglia is required for Aβ clearance upon IL-33 treatment. **a,b**, Chemotactic microglia migrate toward Aβ plaques after IL-33 treatment. Representative images (a) and violin plot (b) showing the distance between chemotactic microglia (that is, *Vcam1*^+^ *Cx3cr1*^+^ cells) and the nearest Aβ plaque 3, 8 and 24 h after IL-33 treatment (3 h: *n* = 83 microglia from four mice; 8 h: *n* = 92 microglia from four mice; 24 h: *n* = 89 microglia from four mice; Kruskal–Wallis test with Dunn’s multiple comparisons test). Dotted circle indicates 10 μm from the perimeter of the Aβ plaque. Arrowheads indicate *Vcam1*-expressing microglia. Scale bar = 10 μm. **c**,**d**, Genetic ablation of ST2 inhibits the microglial chemotactic state 3 h after IL-33 treatment. Representative contour plots (c) and bar plot (d) showing the proportions of chemotactic microglia in each group (Con APP/PS1;ST2WT mice: *n* = 4; IL-33–treated APP/PS1;ST2WT mice: *n* = 5; Con APP/PS1;ST2KO mice: *n* = 4; IL-33–treated APP/PS1 mice; ST2KO: *n* = 5; two-way ANOVA with Šidák’s multiple comparisons test). **e**–**g**, Genetic ablation of ST2 inhibits the induction of MHC-II^+^ phagocytic microglia 24 h after IL-33 treatment. Representative images (e) and bar plots showing the proportions of Aβ plaque-associated microglia (f) and phagocytic microglia (g) in each group (Con APP/PS1;ST2WT mice*:* *n* = 9 for panel f and *n* = 10 for panel g; IL-33-treated APP/PS1;ST2WT mice: *n* = 10 for panel f and *n* = 8 for panel g; Con APP/PS1;ST2KO mice: *n* = 8; IL-33-treated APP/PS1;ST2KO mice: *n* = 9; two-way ANOVA with Šidák’s multiple comparisons test). Arrowheads indicate phagocytic microglia. Scale bar = 20 μm. **h**,**i**, Genetic ablation of ST2 attenuates Aβ clearance induced by IL-33 48 h treatment. Representative images (h) and bar plot (i) showing the Aβ plaque area in the cortex 48 h after IL-33 treatment in each group (Con APP/PS1;ST2WT mice: *n* = 10; IL-33-treated APP/PS1;ST2WT mice: *n* = 9; Con APP/PS1;ST2KO mice: *n* = 9; IL-33–treated APP/PS1;ST2KO mice: *n* = 7; two-way ANOVA with Šidák’s multiple comparisons test). Scale bar = 200 μm. All data are mean ± s.e.m. Source data

**Extended Data Fig. 2:**
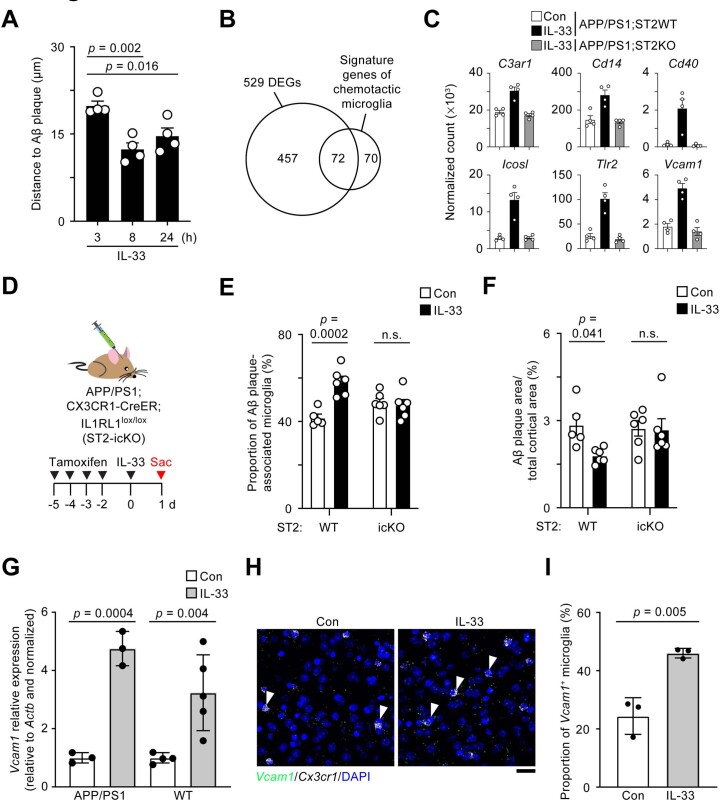
ST2 genetic ablation inhibits the induction of the chemotactic transcriptomic state in microglia after interleukin-33 treatment. **a**, Bar plot showing the distance between chemotactic microglia (that is, *Vcam1*^+^ *Cx3cr1*^+^ cells) and the nearest amyloid-beta (Aβ) plaque 3, 8, and 24 h after interleukin-33 (IL-33) treatment (*n* = 4 mice per condition; one-way ANOVA with Dunnett’s multiple comparisons test). **b**, Venn diagram showing the overlap between 529 differentially expressed genes (DEGs) (IL-33–treated APP;ST2KO vs. IL-33-treated APP;ST2WT mice) and the signature genes of chemotactic microglia (*n* = 4 per condition). **c**, Bar plots showing the expression levels of the representative chemotactic signature genes in the 3 conditions (Wald test from *DESeq2*). **d-f**, Genetic ablation of ST2 in microglia inhibits the Aβ chemotaxis of microglia and microglia-mediated Aβ clearance upon IL-33 treatment. **d**, Schematic diagram showing the protocol for tamoxifen and IL-33 administration in APP/PS1;ST2-icKO mice. **e**,**f**, Bar plots showing the proportions of Aβ plaque-associated microglia (e) and Aβ levels in cortical regions (f) after IL-33 treatment in APP/PS1;ST2-icKO mice (wild-type [WT] Con: *n* = 5; WT IL-33: *n* = 6; icKO Con: *n* = 6; icKO IL-33: *n* = 6; two-way ANOVA with Šidák’s multiple comparisons test). **g-i**, The induction of VCAM1^+^ chemotactic microglia is a generalized IL-33 response in microglia. **g**, Bar plot showing the expression level of *Vcam1* in microglia 3 h after IL-33 treatment (WT Con: *n* = 4; WT IL-33: *n* = 5; APP/PS1 Con: *n* = 3; APP/PS1 IL-33: *n* = 3; two-way ANOVA with Šidák’s multiple comparisons test). **h**,**i**, Representative images (h) and quantification (i) showing the proportion of chemotactic microglia (that is, *Vcam1*^+^ *Cx3cr1*^+^ cells) after interleukin-33 (IL-33) treatment in WT mice (*n* = 3 mice per condition; two-tailed unpaired Student’s *t*-test). Arrowheads indicate *Vcam1*-expressing microglia. Scale bar = 10 μm. All data are mean ± s.e.m. Source data

Next, we investigated how the induction of VCAM1^+^ chemotactic microglia contributes to IL-33-stimulated Aβ clearance. As VCAM1^+^ chemotactic microglia are induced during the chemotactic phase, we first examined whether these microglia are the microglial subpopulation that migrates toward Aβ plaques upon IL-33 treatment. In situ hybridization analysis showed that the distance between *Vcam1*^+^ microglia and the nearest Aβ plaque gradually decreased after IL-33 treatment (Fig. 2a,b and Extended Data Fig. 2a), confirming that these microglia exhibit Aβ-directed chemotaxis.

We then examined how IL-33 stimulates the induction of this VCAM1^+^ chemotactic microglial subpopulation and whether this process is required for IL-33-stimulated Aβ clearance. Accordingly, we first generated APP/PS1 transgenic mice that lacked the IL-33 receptor ST2 (that is, APP/PS1;ST2KO) and examined the effects of IL-33 on microglial state transition and Aβ clearance in these mice. Flow cytometry analysis showed that ST2 genetic ablation inhibited the increase in the proportion of VCAM1^+^ chemotactic microglia that occurs upon IL-33 treatment (Fig. 2c,d). Genetic ablation of ST2 also abolished the induction of the chemotactic gene signature in microglia that occurs after IL-33 treatment in APP/PS1 mice (Extended Data Figs. 2b,c). Importantly, ST2 genetic ablation abolished microglial differentiation into the MHC-II^+^ phagocytic state (Fig. 2e–g) and attenuated Aβ clearance (Fig. 2h,i) after IL-33 stimulation. We further generated microglial ST2 conditional-knockout APP/PS1 mice (that is, APP/PS1;ST2-icKO) and showed that microglial ST2 is required for IL-33-induced microglial Aβ chemotaxis and clearance (Extended Data Fig. 2d–f). Also, IL-33 stimulates *Vcam1* expression in microglia from wild-type mice, suggesting that the induction of VCAM1^+^ chemotactic state in microglia is a generalized IL-33 response that does not require priming (Extended Data Fig. 2g–i). These findings collectively show that ST2 is required for the induction of chemotactic microglia after IL-33 treatment and that ST2-dependent microglial state transition is a prerequisite for IL-33-stimulated Aβ clearance.

### VCAM1 regulates Aβ chemotaxis of IL-33RM

**Fig. 3:**
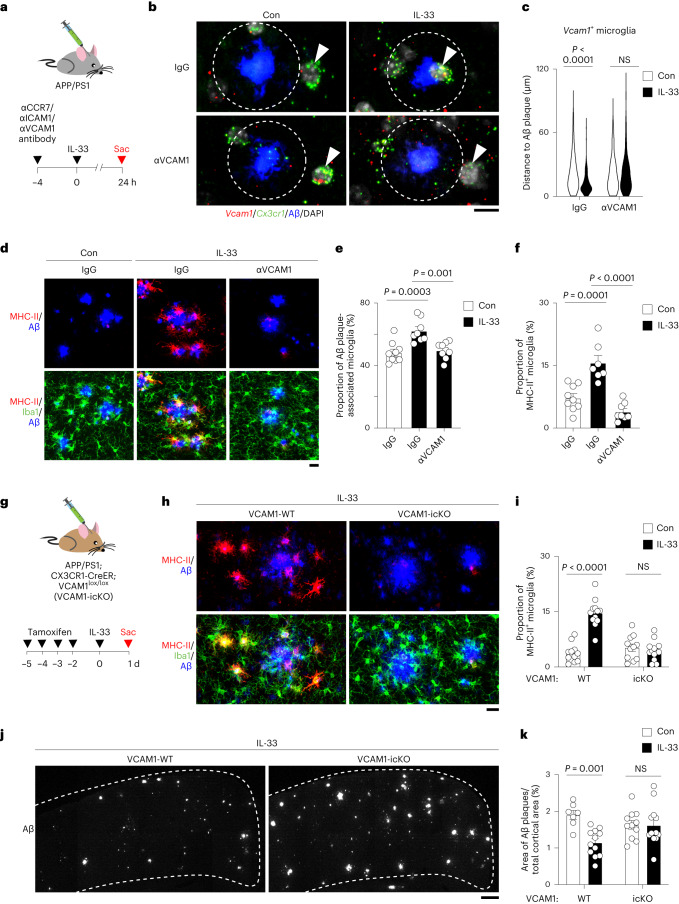
VCAM1 controls the Aβ chemotaxis of chemotactic microglia and subsequent Aβ clearance upon IL-33 treatment. **a–f**, VCAM1 blockade inhibits the IL-33-induced Aβ chemotaxis of microglia. **a,** Schematic diagram showing the protocol for neutralizing antibody administration before IL-33 treatment in APP/PS1 mice. **b**,**c**, Representative images (b) and violin plot (c) showing the distances between chemotactic microglia and the nearest Aβ plaque 24 h after administration of a VCAM1-neutralizing antibody in IL-33-treated APP/PS1 mice (IgG Con: *n* = 61 microglia from 5 mice; IgG IL-33: *n* = 131 microglia from 5 mice; αVCAM1 Con: *n* = 48 microglia from 5 mice; αVCAM1 IL-33: *n* = 109 microglia from 5 mice; Kruskal–Wallis test with Dunn’s multiple comparisons test). Dotted circle indicates 10 μm from the perimeter of the Aβ plaque. Arrowheads indicate *Vcam1*-expressing microglia. Scale bar = 10 μm. **d–f**, Representative images (d) and bar plots showing the proportions of Aβ plaque-associated microglia (e) and phagocytic microglia (f) 24 h after administration of antibody against VCAM1 in IL-33-treated APP/PS1 mice (IgG Con: *n* = 9; IgG IL-33: *n* = 8 for panel e and *n* = 7 for panel f; αVCAM1 IL-33: *n* = 9 for panel e and *n* = 8 for panel f; one-way ANOVA with Dunnett’s multiple comparisons test). Scale bar = 20 μm. **g–k**, Genetic ablation of VCAM1 in microglia inhibits the Aβ chemotaxis of microglia and microglia-mediated Aβ clearance upon IL-33 treatment. **g**, Schematic diagram showing the protocol for tamoxifen and IL-33 administration in APP/PS1;VCAM1-icKO mice. **h**,**i**, Representative images (h) and bar plot (i) showing the proportions of phagocytic microglia 24 h after IL-33 treatment in APP/PS1;VCAM1-icKO mice (wild-type (WT) Con: *n* = 11; WT IL-33: *n* = 12; icKO Con: *n* = 11; icKO IL-33: *n* = 13; two-way ANOVA with Šidák’s multiple comparisons test). Scale bar = 20 μm. **j**,**k**, Representative images (j) and bar plot (k) showing the Aβ plaque areas in the cortex 48 h after IL-33 treatment in APP/PS1-icKO mice (WT Con: *n* = 7; WT IL-33: *n* = 12; icKO Con: *n* = 11; icKO IL-33: *n* = 12; two-way ANOVA with Šidák’s multiple comparisons test). Scale bar = 200 μm. All data are mean ± s.e.m. Source data

**Extended Data Fig. 3:**
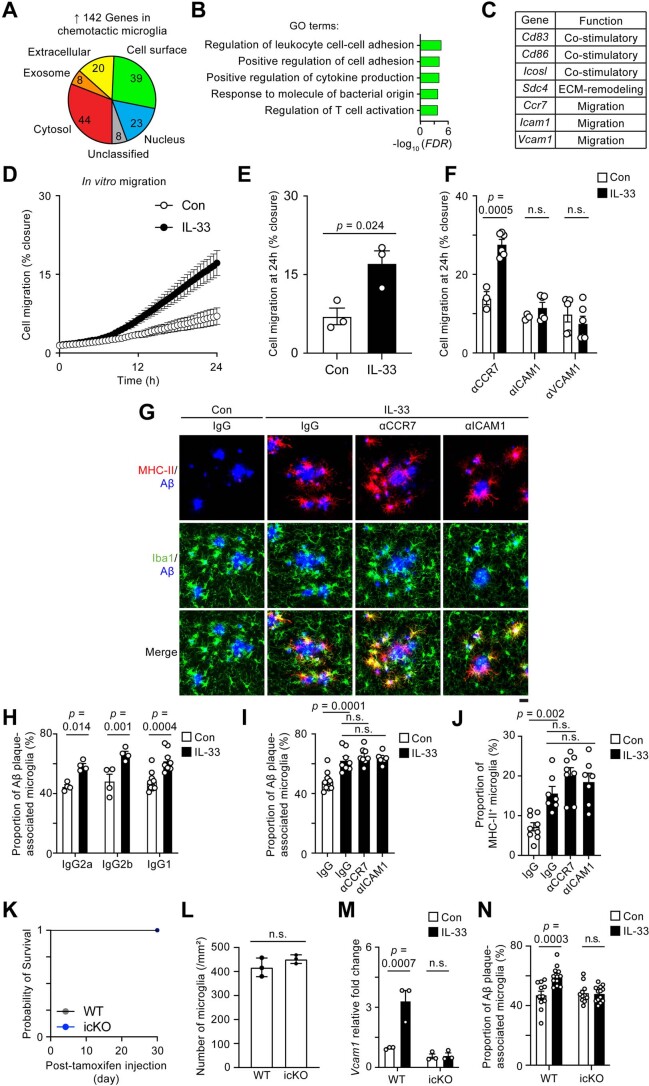
VCAM1 regulates microglial amyloid-beta chemotaxis after interleukin-33 treatment. **a-c**, Identification of a potential cell-surface receptor that controls the amyloid-beta (Aβ) chemotaxis of chemotactic microglia. **a**, Subcellular distribution of the 142 signature genes of chemotactic microglia. **b**, Gene Ontology (GO) analysis of the 39 chemotactic signature genes localized on the cell surface. FDR, false discovery rate. **c**, Functional classification of the 7 cell adhesion molecules. **d-f**, Neutralizing antibodies against ICAM1 and VCAM1 inhibit interleukin-33 (IL-33)–stimulated microglia migration *in vitro*. **d**,**e**, IL-33 promotes the migratory response of BV2 cells in a wound-healing assay (*n* = 3 from 3 independent batches; two-tailed unpaired Student’s *t*-test). **f**, Quantification showing the effects of CCR7-, ICAM1-, and VCAM1-neutralizing antibodies on the IL-33–stimulated migration of BV2 cells in a wound-healing assay (αCCR7 Con: *n* = 3; αCCR7 IL-33: *n* = 6; αICAM1 Con: *n* = 3; αICAM1 IL-33: *n* = 6; αVCAM1 Con: *n* = 5; αVCAM1 IL-33: *n* = 6; two-way ANOVA with Šidák’s multiple comparisons test). **g-k**, Neutralizing antibodies against CCR7, ICAM1, or isotype controls neither affect Aβ chemotaxis nor the induction of MHC-II^+^ microglia after IL-33 treatment.**g**,**h**, Representative images (g)and quantification (h) showing the proportions of Aβ plaque-associated microglia after administration of isotype control antibodies and IL-33 (IgG2a Con: *n* = 4; IgG2a IL-33: *n* = 4; IgG2b Con: *n* = 4; IgG2b IL-33: *n* = 4; IgG1 Con: *n* = 9; IgG1 IL-33: *n* = 8; two-way ANOVA with Šidák’s multiple comparisons test). **i**,**j**, Quantification showing the proportions of Aβ plaque-associated microglia (i) and the proportions of MHC-II^+^ microglia (j) after administration of neutralizing antibodies and IL-33 (IgG Con: *n* = 9; IgG IL-33: *n* = 8 for panel i and 7 for panel j; αCCR7 IL-33: *n* = 8; αICAM1 IL-33: *n* = 7; one-way ANOVA with Šidák’s multiple comparisons test). Scale bar = 20 μm. **k**,**l**, Long-term genetic ablation of microglial VCAM1 does not affect the survival of mice or the number of microglia. **k**, Survival curve of VCAM1-icKO mice 1 month after tamoxifen injection (*n* = 3 per condition). **l**, Quantification showing the number of microglia in VCAM1-icKO mice (*n* = 3 per condition; two-tailed unpaired Student’s *t*-test). **m**, Genetic ablation of microglial VCAM1 abolishes IL-33–induced *Vcam1* expression in microglia (wild-type [WT] Con: *n* = 3; WT IL-33: *n* = 3; icKO Con: *n* = 3; icKO IL-33: *n* = 3; two-way ANOVA with Šidák’s multiple comparisons test). **n**, Genetic ablation of microglial VCAM1 inhibits microglial migration toward Aβ plaques after IL-33 treatment. Quantification showing the proportions of Aβ plaque-associated microglia in VCAM1-icKO mice after IL-33 treatment (WT Con: *n* = 11; WT IL-33: *n* = 12; icKO Con: *n* = 11; icKO IL-33: *n* = 13; two-way ANOVA with Šidák’s multiple comparisons test). All data are mean ± s.e.m. Source data

During chemotaxis, cell-surface receptors sense chemoattractants to regulate cell migration^24,25^. Therefore, we investigated which cell-surface receptor in chemotactic microglia mediates Aβ chemotaxis. Compared to other microglial subpopulations, chemotactic microglia exhibited higher expression of 39 cell-surface receptors (Extended Data Fig. 3a); 3 of these receptors (*Ccr7*, *Icam1* and *Vcam1*) are functionally associated with the cell migration response (Extended Data Fig. 3b,c). To investigate whether their respective encoded cell-surface receptors are important for the enhanced chemotactic capacity of VCAM1^+^ chemotactic microglia, we performed an in vitro wound-healing migratory assay using the BV2 microglial cell line. Only neutralizing antibodies against ICAM1 and VCAM1 inhibited the IL-33-stimulated migration capacity of BV2 cells (Extended Data Fig. 3d–f). Next, we treated APP/PS1 mice with neutralizing antibodies targeting each of the receptors before IL-33 treatment (Fig. 3a). Only VCAM1-neutralizing antibody and not antibodies against CCR7 and ICAM1 or their isotype control antibodies inhibited the IL-33-induced chemotactic microglial migration toward Aβ plaques (Fig. 3b,c and Extended Data Fig. 3g–i). Moreover, only VCAM1-neutralizing antibody attenuated the subsequent transition of microglia into the MHC-II^+^ phagocytic state after IL-33 treatment (Fig. 3d–f and Extended Data Fig. 3j).

To examine whether VCAM1-mediated signaling in microglia is important for IL-33-induced Aβ chemotaxis, we generated VCAM1 conditional-knockout APP/PS1 mice (that is, APP/PS1;VCAM1-icKO) and examined the effect of IL-33 on microglial Aβ chemotaxis (Fig. 3g). Genetic ablation of microglial VCAM1 abolishes *Vcam1* expression in microglia after IL-33 treatment but does not affect the survival of mice or the number of microglia in cortical regions (Extended Data Fig. 3k–m). Next, we showed that genetic ablation of microglial VCAM1 attenuated the IL-33-induced increase in the number of Aβ plaque-associated microglia (Fig. 3h and Extended Data Fig. 3n), suggesting that microglial VCAM1 is important for IL-33-stimulated microglial migration toward Aβ plaques. Moreover, in the APP/PS1;VCAM1-icKO mice, IL-33 stimulated neither the transition of microglia to the MHC-II^+^ phagocytic state (Fig. 3i) nor Aβ clearance (Fig. 3j,k). These findings collectively demonstrate that microglial VCAM1 plays a role in controlling microglial chemotaxis toward Aβ plaques as well as the subsequent IL-33-stimulated Aβ clearance.

### ApoE acts as chemoattractant for VCAM1-dependent chemotaxis

**Fig. 4:**
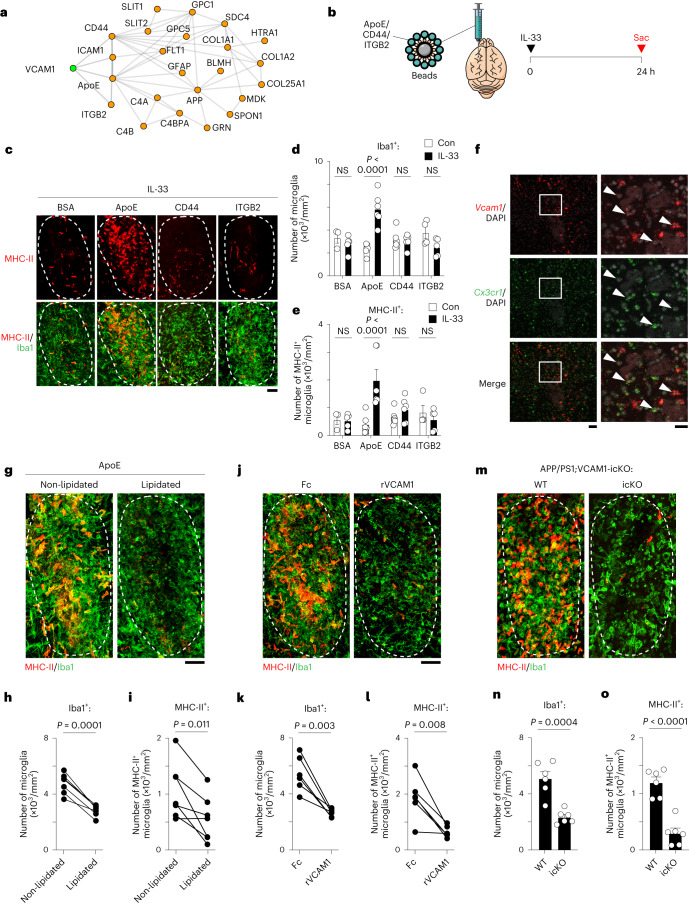
ApoE is a chemoattractant that directs VCAM1-dependent chemotaxis of microglia. **a**, STRINGdb protein–protein interaction between VCAM1 (green) and Aβ plaque-associated proteins (orange). **b**, Schematic diagram showing the protocol for injecting protein-coated beads followed by IL-33 treatment in APP/PS1 mice. **c–e**, Representative images (c) and bar plots showing the numbers of microglia (d) and MHC-II^+^ microglia (e) within the area of BSA-, ApoE-, CD44-, and ITGB2-coated beads after IL-33 treatment (Con: BSA: *n* = 3, ApoE: *n* = 6, CD44: *n* = 6, ITGB2: *n* = 4; IL-33: BSA: *n* = 5, ApoE: *n* = 6, CD44: *n* = 6, ITGB2: *n* = 5; two-way ANOVA with Šidák’s multiple comparisons test). Dotted line indicates bead area. Scale bar = 20 μm. **f**, Representative images showing the *Vcam1*-expressing microglia surrounding ApoE-coated beads after IL-33 treatment. Arrowheads indicate *Vcam1*-expressing microglia. Scale bars = 50 μm (left) and 20 μm (right). Experiment was repeated for three batches with similar results. **g**–i, Representative images (g) and bar plots showing the numbers of microglia (h) and MHC-II^+^ microglia (i) within the areas of beads coated with nonlipidated or lipidated ApoE in IL-33-treated APP/PS1 mice (nonlipidated: *n* = 7; lipidated: *n* = 7; two-tailed paired *t*-test). Dotted line indicates bead area. Scale bar = 20 μm. **j–o**, VCAM1 is essential for the ApoE chemotaxis of microglia after IL-33 treatment. **j–l**, Representative images (j) and bar plots showing the numbers of microglia (k) and MHC-II^+^ microglia (l) within the ApoE-coated bead areas after co-injection of rVCAM1 in IL-33-treated APP/PS1 mice (Fc: *n* = 6; rVCAM1: *n* = 6; two-tailed paired *t*-test). Dotted line indicates bead area. Scale bar = 20 μm. **m**–**o**, Representative images (m) and bar plots showing the numbers of microglia (n) and MHC-II^+^ microglia (o) within the ApoE-coated bead areas in IL-33-treated APP/PS1;VCAM1-icKO mice (wild-type [WT]: *n* = 6; icKO: *n* = 6; two-tailed unpaired *t*-test). Dotted line indicates bead area. Scale bar = 20 μm. All data are mean ± s.e.m. Source data

**Extended Data Fig. 4:**
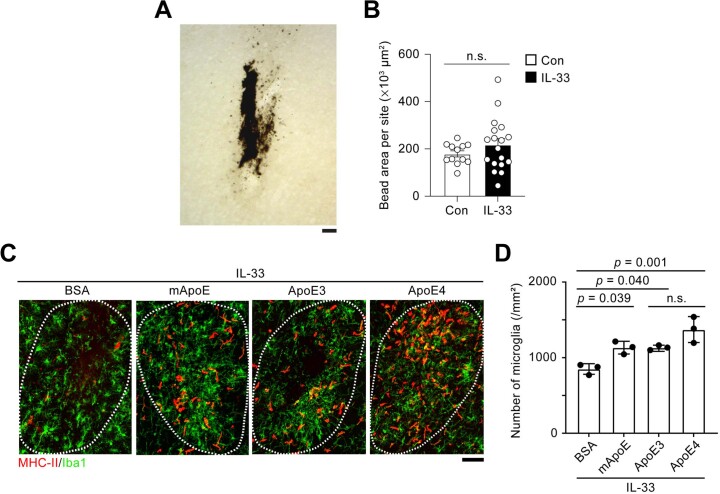
Chemotactic microglia migrate toward nonlipidated ApoE after interleukin-33 treatment. **a**,**b**, Quality control for bead injection. Representative images (a) and quantification (b) showing the bead injection area (Con: *n* = 12; interleukin-33 [IL-33]: *n* = 18, two-tailed unpaired Student’s *t*-test). Scale bar = 50 μm. **c**,**d**, IL-33-induced chemotactic microglia migrate towards human ApoE isoforms. Representative images (c) and bar plot (d) showing microglial migration towards BSA-, murine ApoE (mApoE)-, human ApoE3-, and human ApoE4-coated beads after IL-33 treatment (*n* = 3 per condition; one-way ANOVA with Šidák’s multiple comparisons test). Dotted line indicates bead area. Scale bar = 25 μm. All data are mean ± s.e.m. Source data

We subsequently determined which chemoattractant in Aβ plaques directs VCAM1-dependent chemotaxis in microglia. Although Aβ is the major component of Aβ plaques, these plaques are also enriched with more than 20 proteins, including lipoproteins and truncated receptors^26^. STRING protein–protein interaction analysis revealed that some Aβ plaque-associated proteins (namely ApoE, CD44, ICAM1 and ITGB2) can interact with VCAM1 (Fig. 4a). To determine whether these proteins serve as chemoattractants for VCAM1-dependent microglial chemotaxis, we stereotactically injected APP/PS1 mice with beads coated with recombinant ApoE, CD44 or ITGB2 protein and examined microglial migration 24 h after IL-33 injection, when the microglia had finished migrating toward the beads (Fig. 4b and Extended Data Fig. 4a,b). IL-33 administration increased both the total and MHC-II^+^ microglia surrounding the ApoE-coated beads (Fig. 4c–e). However, we observed no such increase in the total or MHC-II^+^ microglia surrounding the BSA- (as a control), CD44- or ITGB2-coated beads after IL-33 treatment. Also, IL-33-stimulated microglia migrate toward both human ApoE isoforms and murine ApoE to a similar extent (Extended Data Fig. 4c,d). Furthermore, the microglia surrounding the ApoE-coated beads in IL-33-treated APP/PS1 mice expressed *Vcam1* (Fig. 4f), suggesting that ApoE acts as a chemoattractant for the IL-33-induced VCAM1^+^ chemotactic microglia.

ApoE lipidation strongly affects the interaction between ApoE and cell-surface receptors^27–30^. Therefore, we investigated whether ApoE lipidation regulates IL-33-stimulated microglial chemotaxis by stereotactically injecting APP/PS1 mice with lipidated or nonlipidated ApoE. After IL-33 treatment in APP/PS1 mice, more microglia surrounded nonlipidated ApoE than lipidated ApoE (Fig. 4g–i). Blockade of VCAM1 signaling by co-injection of recombinant VCAM1 protein or genetic ablation of VCAM1 in microglia inhibited microglial migration toward nonlipidated ApoE in APP/PS1 mice after IL-33 treatment (Fig. 4j–o). Together, these findings indicate that nonlipidated ApoE preferentially directs the IL-33-induced VCAM1-dependent chemotaxis in microglia.

### VCAM1–ApoE interaction controls microglial Aβ chemotaxis

**Fig. 5:**
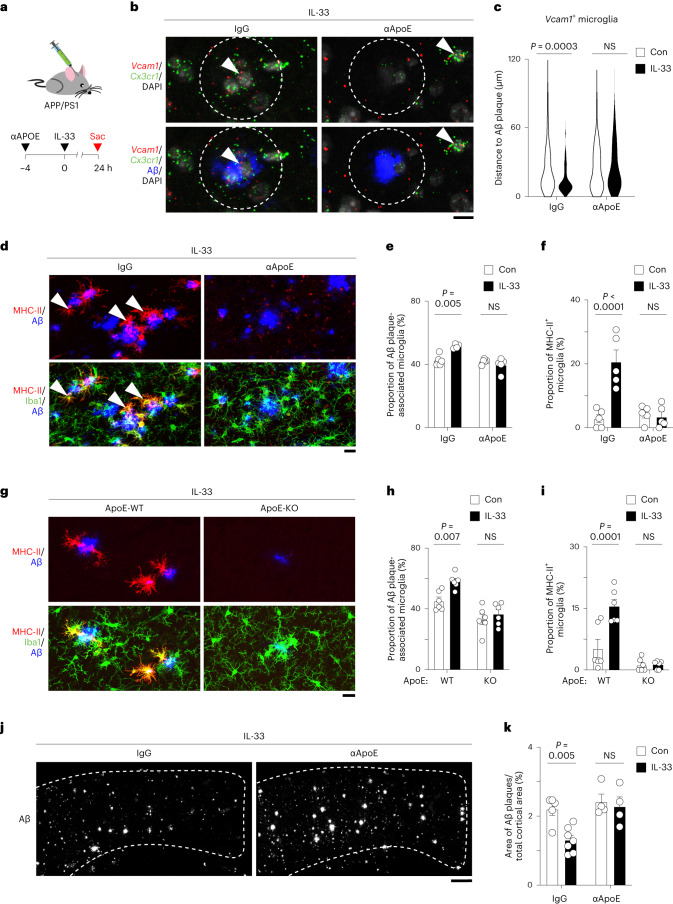
VCAM1–ApoE interaction is critical for the Aβ chemotaxis of microglia and microglia-mediated Aβ clearance after IL-33 treatment. **a–c**, ApoE-neutralizing antibody inhibits the Aβ chemotaxis of microglia upon IL-33 treatment. **a**, Schematic diagram showing the protocol for ApoE-neutralizing antibody administration before IL-33 treatment in APP/PS1 mice. **b**,**c**, Representative images (b) and violin plot (c) showing the distance between chemotactic microglia (that is, *Vcam1*^+^ *Cx3cr1*^+^ cells) and the nearest Aβ plaque 24 h after administration of ApoE-neutralizing antibody in IL-33-treated APP/PS1 mice (IgG Con: *n* = 107 microglia from 6 mice; IgG IL-33: *n* = 115 microglia from 5 mice; αApoE Con: *n* = 94 microglia from 6 mice; αApoE IL-33: *n* = 93 microglia from 6 mice; Kruskal–Wallis test with Dunn’s multiple comparisons test). Dotted circle indicates 10 μm from the perimeter of the Aβ plaque. Arrowheads indicate *Vcam1*-expressing microglia. Scale bar = 10 μm. **d**–**i**, VCAM1–ApoE interaction is required for inducing the phagocytic state transition of microglia after the induction of VCAM1 expression. **d**–**f**, Representative images (d) and bar plots showing the proportions of Aβ plaque-associated microglia (e) and phagocytic microglia (f) (that is, MHC-II^+^ Iba1^+^ cells) 24 h after administration of ApoE-neutralizing antibody in IL-33-treated APP/PS1 mice (IgG Con: *n* = 5; IgG IL-33: *n* = 5; αApoE Con: *n* = 5; αApoE IL-33: *n* = 5; two-way ANOVA with Šidák’s multiple comparisons test). Arrowheads indicate phagocytic microglia. Scale bar = 20 μm. **g**–**i**, Representative images (g) and bar plots showing the proportions of Aβ plaque-associated microglia (h) and phagocytic microglia (i) (that is, MHC-II^+^ Iba1^+^ cells) 24 h after IL-33 treatment in APP/PS1-ApoE–knockout mice (*n* = 6 per condition; two-way ANOVA with Šidák’s multiple comparisons test). Scale bar = 20 μm. **j**,**k**, Representative images (j) and bar plot (k) showing the Aβ plaque areas in the cortex 48 h after administration of ApoE-neutralizing antibody in IL-33-treated APP/PS1 mice (IgG Con: *n* = 5; IgG IL-33: *n* = 7; αApoE Con: *n* = 4; αApoE IL-33: *n* = 4; two-way ANOVA with Šidák’s multiple comparisons test). Scale bar = 200 μm. All data are mean ± s.e.m. Source data

In the AD brain, ApoE is secreted by DAM, astrocytes, vascular cells, and stressed neurons^13,30,31^. Secreted ApoE can bind to Aβ, thereby facilitating its seeding and enhancing the compactness of Aβ plaques^32–34^. Interestingly, Aβ plaque-associated ApoE is predominantly nonlipidated^35^. Therefore, we investigate whether ApoE associated with Aβ plaques is a major mediator of the migration of chemotactic microglia toward Aβ plaques. Accordingly, we administered ApoE-neutralizing antibody to APP/PS1 mice to inhibit microglia–ApoE interaction^36^ before IL-33 treatment (Fig. 5a). Blockade of ApoE signaling by this antibody inhibited the migration of VCAM1^+^ chemotactic microglia toward Aβ plaques after IL-33 treatment (Fig. 5b,c). Moreover, the antibody abolished the subsequent increase in the proportion of Aβ plaque-associated microglia and MHC-II^+^ phagocytic microglia stimulated by IL-33 (Fig. 5d–f). To further examine the role of ApoE in the regulation of VCAM1-dependent microglial chemotaxis, we generated ApoE-knockout APP/PS1 mice (that is, APP/PS1;ApoE-KO) and examined the effect of IL-33 on microglial Aβ chemotaxis. Genetic ablation of ApoE inhibited the recruitment of microglia toward Aβ plaques and the subsequent induction of MHC-II^+^ phagocytic microglia after IL-33 treatment (Fig. 5g–i). These results suggest that VCAM1–ApoE interaction is essential for directing chemotactic microglia toward Aβ plaques and the transition of these microglia to the subsequent phagocytic state. Consistent with this notion, the ApoE-neutralizing antibody also abolished the IL-33-induced Aβ clearance (Fig. 5j,k). Although previous report suggested that chronic inhibition of ApoE for 14 weeks reduces Aβ levels in APP/PS1 mice^36^, we did not observe notable reduction in Aβ level after acute ApoE inhibition (that is, within 52 h). Together, these findings demonstrate that the interaction between VCAM1^+^ chemotactic microglia and Aβ plaque-associated ApoE is required for microglia to transition from a chemotactic state to a phagocytic state and for subsequent Aβ clearance.

### sVCAM1 correlated with microglia–Aβ interaction in AD

**Extended Data Fig. 5:**
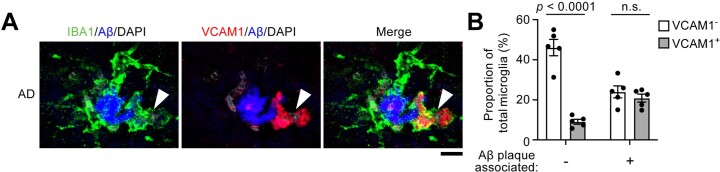
VCAM1^+^ microglia interact with amyloid-beta plaques in the brains of patients with Alzheimer’s disease. **a**,**b**, Representative images (a) and bar plot (b) showing the proportions of amyloid-beta (Aβ) plaque-associated VCAM1^+^ microglia in postmortem Alzheimer’s disease brain sections (*n* = 5 patients with Alzheimer’s disease; two-way ANOVA with Šidák’s multiple comparisons test). Arrowheads indicate the VCAM1^+^ microglia associated with plaque. Scale bar = 10 μm. All data are mean ± s.e.m. Source data

Although the VCAM1–ApoE axis is important for IL-33-stimulated microglial chemotaxis toward Aβ plaques and subsequent Aβ clearance, it is unclear if patients with AD have impaired microglial VCAM1 signaling. Accordingly, we examined the microglia in the brains of patients with AD; 29.9% of microglia expressed VCAM1, 69.7% of which (that is, 20.9% of the total microglia) were co-localized with Aβ plaques (Extended Data Fig. 5a,b). These findings show that most VCAM1^+^ microglia interact with Aβ plaques in patients with AD.

**Fig. 6:**
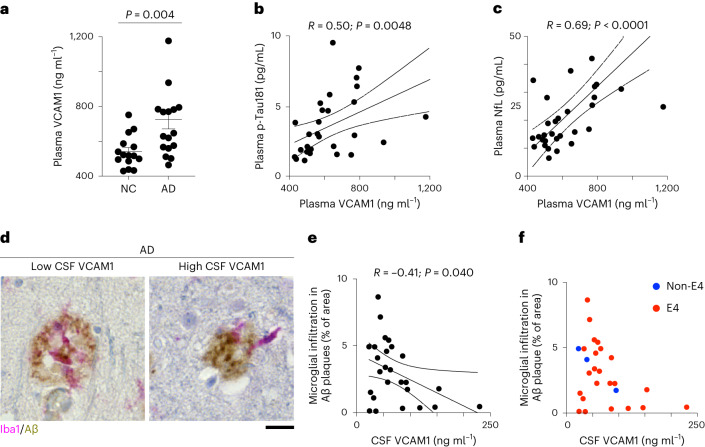
Dysregulated VCAM1 signaling is associated with impaired microglial infiltration into Aβ plaques in patients with AD. **a–c**, Soluble VCAM1 (sVCAM1) level is elevated in the plasma of patients with AD and correlated with disease severity. **a**, sVCAM1 levels in the plasma of normal controls (NCs) and patients with AD (NC: *n* = 15; AD: *n* = 17; two-tailed Mann–Whitney test). **b**,**c**, Correlations between plasma levels of sVCAM1 and plasma p-Tau181 (b) (tau phosphorylated at threonine-181) and plasma NfL (c) (neurofilament light polypeptide) (*n* = 30 for panel b, *n* = 31 for panel c; linear regression). Dotted line indicates the 95% confidence interval of the regression line. **d**–**f**, Cerebrospinal fluid (CSF) sVCAM1 levels are inversely correlated with microglial infiltration into Aβ plaques. Representative images (d) and dot plot (e) showing the correlation between CSF sVCAM1 level and microglial infiltration into Aβ plaques in patients with AD (*n* = 26, linear regression). Dotted line indicates the 95% confidence interval of the regression line. Scale bar = 20 μm. **f**, Dot plot showing the correlations between CSF sVCAM1 level and microglial infiltration into Aβ plaques in patients with AD stratified by ApoE4 genotype. All data are mean ± s.e.m. Source data

**Extended Data Fig. 6:**
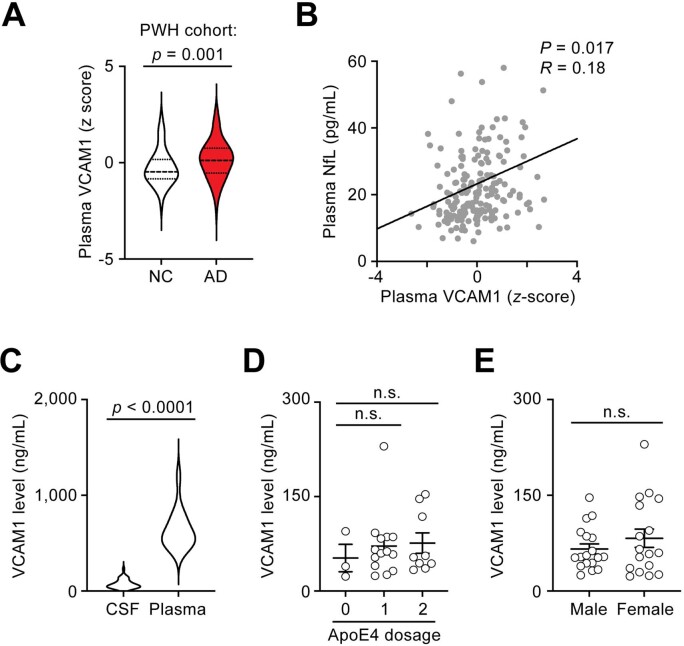
Validation of the elevated plasma soluble VCAM1 level in patients with Alzheimer’s disease. **a**,**b**, Cross-cohort validation of the elevated plasma soluble VCAM1 (sVCAM1) level found in patients with Alzheimer’s disease (AD). **a**, Violin plots showing the levels of plasma sVCAM1 in the PWH cohort (obtained from Jiang et al., 2021) (normal controls [NCs]: *n* = 74; AD: *n* = 106; two-tailed Mann–Whitney test). **b**, Correlation between the plasma levels of sVCAM1 and neurofilament light polypeptide (NfL) (linear regression). **c-e**, Cerebrospinal fluid (CSF) sVCAM1 level is not correlated with sex or *APOE4* genotypes in patients with AD. **c**, Violin plot comparing sVCAM1 levels in CSF and plasma (CSF: *n* = 35; Plasma: *n* = 32; two-tailed Mann–Whitney test). **d**,**e**, Bar plots showing the effects of *APOE4* gene dosage (e) (0: *n* = 3; 1: *n* = 14; 2: *n* = 9; Kruskal–Wallis test with Dunn’s multiple comparisons test) and sex (e) on sVCAM1 level in patients with AD (male: *n* = 18; female: *n* = 17; two-tailed Mann–Whitney test). Data are mean ± s.e.m. Source data

Next, we determined if VCAM1 signaling is dysregulated in patients with AD. The plasma level of soluble VCAM1 (sVCAM1), which acts as a decoy receptor that inhibits VCAM1-mediated signaling, was 34% higher in patients with AD than in healthy controls (Fig. 6a). Moreover, plasma sVCAM1 level was positively correlated with the levels of plasma p-Tau181 (tau phosphorylated at threonine-181) and plasma NfL (neurofilament light polypeptide), which are AD biomarkers that can indicate disease stage (Fig. 6b,d). Consistently, this elevated plasma sVCAM1 level in AD and its correlation with plasma p-Tau181 level were also observed when subjecting the same samples to proximity extension assay proteomic measurement (Extended Data Fig. 6a,b)^37^.

Given the important role of VCAM1 signaling in mediating microglial Aβ chemotaxis, we determined if sVCAM1 level is associated with dysregulated microglia–Aβ interaction in the brain sections of patients with AD. The cerebrospinal fluid (CSF) level of sVCAM1 was inversely correlated with microglial infiltration into Aβ plaques in the brains of patients with AD (Fig. 6d,e and Extended Data Fig. 6c–e); moreover, this inverse correlation was independent of *APOE4* genotype (Fig. 6f). Together, these findings demonstrate that an elevated brain sVCAM1 level is correlated with impaired microglia–Aβ plaques interaction in patients with AD.

## Discussion

In this study, we revealed the critical axes of receptor–ligand interaction that drives Aβ clearance in microglia. The sequential orchestration of the IL-33–ST2 and VCAM1–ApoE pathways is important for promoting Aβ chemotaxis in microglia and ultimately results in Aβ clearance. To stimulate Aβ chemotaxis, IL-33–ST2 signaling first induces the VCAM1^+^ chemotactic state in microglia. Then, induced VCAM1 receptors interact with Aβ plaque-associated ApoE to regulate Aβ-directed migration. Furthermore, our findings reveal an unexpected role of Aβ plaque-associated ApoE whereby it functions as a chemoattractant to direct microglial migration. This study is one of the first to show that the interaction between a microglial receptor and Aβ plaque-associated factor controls Aβ chemotaxis in microglia and leads to Aβ clearance. These findings also suggest the therapeutic potential of targeting these regulatory receptor–ligand axes of microglial chemotaxis to ameliorate AD pathology.

Our study identifies the role of VCAM1 in the regulation of immune cell chemotaxis. Previous studies identified VCAM1 as a cytokine-inducible molecule in endothelial cells that regulates their binding with immune cells^38,39^. Indeed, in various inflammatory conditions, endothelial VCAM1 is induced to prime the site for leukocyte transmigration^40–42^. These findings show that endothelial VCAM1 regulates chemotaxis by functioning as a molecular tag (that is, as a ligand for chemotaxis) to provide directionality for migratory immune cells. Interestingly, VCAM1 can also be induced in migratory immune cells including microglia and macrophages^43–45^. However, it is unclear whether VCAM1 plays functional roles in these immune cells. Here, we found that when VCAM1 is induced in migratory cells (for example, chemotactic microglia), it functions as a chemotactic receptor to sense a chemoattractant(s). These results show that VCAM1 can regulate chemotaxis in a reciprocal manner—acting as a receptor or ligand—depending on the type of cell on which it is expressed. Taken together, the current findings advance our understanding of the role of VCAM1 in the regulation of chemotaxis.

The identification of VCAM1 as a cytokine-induced chemotactic receptor in microglia provides insights into the regulatory principles of microglial chemotaxis in AD. In AD, DAMP accumulation creates a chemoattractant gradient that guides microglial chemotaxis. Successful chemotaxis enables microglia to migrate toward DAMPs and subsequently triggers contact-based phagocytic clearance. Therefore, microglial chemotaxis is an essential intermediate process in DAMP clearance that bridges the functional transition from surveillance to phagocytosis^3,6,7^. Indeed, proper microglial chemotaxis is crucial for limiting the development of amyloid pathology. In particular, genetic ablation of the chemokine receptor CCR2 impairs microglial migration toward Aβ plaques and exacerbates amyloid pathology^46,47^, whereas IL-3 or IL-33 stimulates microglial Aβ chemotaxis to ameliorate amyloid pathology^23,48,49^. These findings also suggest that microglial chemotaxis requires stimulation from extrinsic signals including cytokines and chemokines. However, it is poorly understood how microglia modify their chemotactic capacity and migrate specifically toward Aβ plaques. Our study demonstrates that upon extrinsic signal stimulation, surface receptors are induced to sense the chemoattractant and thereby control the directionality of microglial chemotaxis. These findings demonstrate that both receptor-directed migration and extrinsic signal stimulation are essential for successful microglial chemotaxis in AD.

In addition to the regulation of microglial chemotaxis, surface receptors are important for the functional transition of microglia in AD. Indeed, proper functioning of surface receptors (for example, TREM2 and LRP1) is crucial for interactions between DAMPs and microglia, including phagocytosis, barrier formation, and degradation^20,34,50–54^. These findings collectively demonstrate that receptor–DAMP interaction regulates multiple processes during microglial DAMP clearance, including chemotaxis, phagocytosis, and degradation. Nevertheless, it is unclear how microglia modify their response to DAMPs and transition between functions during DAMP clearance. Although detailed investigations are required to fill this knowledge gap, our single-cell transcriptomic profiling showed that microglia undergo stepwise transcriptomic reprogramming during DAMP clearance. Therefore, we hypothesize that microglia modify their functions by expressing different surface receptors during DAMP clearance. For example, when microglia are in the chemotactic state, the induced VCAM1 receptor interacts with ApoE, which may result in the activation of downstream Rac1 signaling to stimulate cytoskeleton remodeling^24,25,55,56^. Meanwhile, in the phagocytic state, microglia express a distinct set of surface receptors (for example, Trem2) through which they interact with DAMPs and subsequently trigger downstream signaling to stimulate phagocytosis^20,29,57–60^. Accordingly, further studies are required to investigate the potential crosstalk between VCAM1–ApoE signaling and other signal pathways such as TREM2–ApoE. Nevertheless, these findings suggest that by changing the expression profiles of surface receptors, microglia modify the functional outcomes of their interaction with DAMPs, resulting in a functional transition during DAMP clearance.

What drives the induction of the phagocytic state in microglia? Although the activation of surface VCAM1 receptor triggers varies downstream signals including Rac1–Pak1 signaling and calcium-mediated signaling^55,56,61^, further investigation is needed to determine whether VCAM1 activation triggers transcriptional changes in microglia and leads to the induction of the phagocytic signature. Another possibility is that an additional receptor–ligand pair(s) is involved in controlling the transcriptional reprogramming from the chemotactic state to the phagocytic state in microglia. For example, another receptor in chemotactic microglia downstream of VCAM1–ApoE-signaling may interact with its cognate ligand present on Aβ plaques to trigger the expression of the phagocytic signature. Indeed, some signature genes of chemotactic microglia, including *Marco* and *Tlr2*, bind with Aβ to trigger microglial activation^12,62^. Therefore, detailed investigations are required to further dissect the receptor–ligand pair(s) that controls the microglial transition from the chemotactic state to the phagocytic state.

Our model of the microglial homeostatic–chemotactic–phagocytic state transition may be a generalized paradigm for the regulation of microglial functions in AD, partly owing to the presence of VCAM1^+^ and MHC-II^+^ microglia in patients with AD^63–67^. Interestingly, the two key regulatory axes of microglial chemotaxis—IL-33–ST2 and VCAM1 signaling—are dysregulated in AD and aging^49,68,69^. Specifically, the levels of soluble ST2 and sVCAM1, which are inhibitory decoy receptors of ST2 and VCAM1, respectively, are elevated in the CSF and plasma of patients with AD. Therefore, it is of interest to understand how the dysregulation of IL-33–ST2 and VCAM1 signaling impairs microglial chemotaxis in AD. Given that these signaling axes regulate distinct aspects of microglial chemotaxis, the elevated soluble ST2 and sVCAM1 levels in AD may impair the induction of chemotactic microglia and their detection of ApoE in AD, respectively. Indeed, the negative correlation between CSF sVCAM1 level and microglia–Aβ interaction supports the inhibitory role of sVCAM1 on microglial Aβ chemotaxis. Furthermore, these findings show that proper VCAM1 functioning is important for controlling microglial Aβ chemotaxis in patients with AD.

Understanding the molecular mechanisms of neuroprotective functions in microglia, especially DAMP clearance, is pivotal for the development of AD therapeutic interventions. Accordingly, our study identified a VCAM1–APOE pathway that drives Aβ-directed migration in chemotactic microglia and leads to the clearance of Aβ plaques. Therefore, our findings provide valuable therapeutic insights for targeting microglial chemotaxis to ameliorate AD pathology.

## Methods

### Experimental model and subject characteristics

We performed all animal experiments in accordance with protocols A19054 and V190021, which were approved by the Animal Care Committee of the Hong Kong University of Science and Technology (HKUST). We housed all mice at the Laboratory Animal Facility in HKUST. We housed mice of the same sex in temperature and humidity-controlled environment, on a 12-h light/dark cycle and provided them with food and water *ad libitum*. We used mice of both sexes for experiments and performed all experiments using groups of sex- and age-matched (that is, 10–11-month-old) littermates. Consistent with previous findings^23,49,70–72^, we did not observe an obvious sex-specific microglial response toward IL-33.

We obtained four mouse strains from the Jackson Laboratory: APP/PS1 transgenic mice (B6C3-Tg[APPswe, PSEN1dE9]85Dbo), which were generated by incorporating a human/murine APP construct bearing the Swedish double mutation and exon 9-deleted PSEN1 mutation; ApoE-knockout mice (B6.129P2-Apoe^tm1Unc^/J); Cx3cr1^creERT2^ mice (B6.129P2[Cg]-Cx3cr1^tm2.1[cre/ERT2]Litt^) in which a microglia-specific promoter controls CreERT2 expression; and Vcam1^loxP/loxP^ mice (B6.129[C3]-Vcam1^tm2Flv^/J), which have *loxP* sites on either side of the cytokine-responsive promoter region and exon 1 of the *Vcam1* gene. Il1rl1^*loxP/loxP*^ mice, which have *loxP* sites on intron 3 and intron 5, were purchased from GemPharmatech. A.N.J. McKenzie of the Medical Research Council Laboratory of Molecular Biology (Cambridge, UK) provided ST2-deficient mice^73^. We confirmed the genotypes of the mice by PCR analysis of ear biopsy specimens.

The patient study was approved by the Clinical Research & Ethics Committees of Joint Chinese University of Hong Kong-New Territories East cluster for Prince of Wales Hospital (CREC reference no. 2015.461), Kowloon Central Cluster/Kowloon East Cluster for Queen Elizabeth Hospital (KC/KE-15-0024/FR-3) and Human Participants Research Panel of the Hong Kong University of Science and Technology (CRP#180 and CRP#225). All participants provided written informed consent for both study participation and sample collection. We collected plasma samples from healthy controls of Hong Kong Chinese descent and patients with AD aged ≥60 years who visited the Specialist Outpatient Department of the Prince of Wales Hospital at the Chinese University of Hong Kong from April 2013 to February 2018. The clinical diagnosis of AD was based on the criteria for AD in the DSM-5 (Diagnostic and Statistical Manual of Mental Disorders, Fifth Edition). All participants underwent medical history assessment, clinical assessment, cognitive and functional assessments using the Montreal Cognitive Assessment (MoCA), and neuroimaging by magnetic resonance imaging. We excluded participants with any neurological disease other than AD or any psychiatric disorder. We recorded participants’ age, sex, years of education, medical history, history of cardiovascular disease (that is, heart disease, hypertension, diabetes mellitus, and hyperlipidemia), and white blood cell counts. This Chinese cohort data was previously collected and published^74^. In brief, the cohort consists of 345 patients with AD and 345 health controls, and we selected 32 samples (males (M) = 7, females (F) = 25; NC = 15, AD = 17; age = 67–87 years; MoCA = 4–30) for plasma ELISA analysis.

We obtained postmortem formalin-fixed, paraffin-embedded brain sections and cerebrospinal fluid (CSF) samples from patients with AD from the South West Dementia Brain Bank (SWDBB), which receives approval from North Somerset and South Bristol Research Ethics Committee to operate as a research tissue bank (REC reference number: 23/SW/0023). Consent was obtained from potential donors whilst living and the capacity to make this decision at the time of registration was witnessed by an appropriate person in a Qualifying Relationship. In the event that a potential donor no longer had the capacity to consent for themselves, the SWDBB will also accept applications submitted on their behalf by an appropriate person in a Qualifying Relationship under specific conditions. The clinical diagnosis of AD was based on the DSM-5 criteria for AD. For our initial sample selection from the SWDBB, we excluded participants with neurodegenerative diseases other than AD, vascular diseases, an intoxicated state or infection at the time of death, prions, inflammatory diseases, structural brain disorders, metabolic/nutritional diseases, trauma, delirium, genetic disorders (for example, Down syndrome) or systemic diseases other than AD. We selected 35 AD samples (M = 18, F = 17; age = 54-96 years) and the detailed population, including age, sex, APOE4 genotype, CSF VCAM1 level and microglia–Aβ interaction, are shown in Supplementary Table 1. Datapoints with 1) CSF VCAM1 level below detection level, 2) incomplete *APOE4* genotype information and 3) poor immunohistochemical staining of microglia and Aβ were excluded from the analysis in Fig. 6e and Extended Data Fig. 6d. Data distribution was assumed to be normal, but this was not formally tested.

### Reagents

We obtained murine recombinant IL-33 (580506), AF647-conjugated Aβ (clone: 6E10) antibody (803021), APC-conjugated MHC-II (clone: M5/114.15.2) antibody (107614), FITC-conjugated VCAM1 (clone: MVCAM.A) antibody (105706) and MHC-II (I-A/I-E) (clone: M5/114.15.2) antibody (107601) from BioLegend. We obtained ICAM1-neutralizing (clone: YN1/1.7.4) antibody (BE0020)^75,76^ and VCAM1-neutralizing (clone: M/K-2.7) antibody (BE0027)^69,77^ from Bio X Cell. We obtained AF488-conjugated CD11b (clone: M1/70) antibody (53-0112-82), APC-conjugated CD11b (clone: M1/70) antibody (17-0112-83) and biotinylated CD11b (clone: M1/70) antibody (13-0112-82) from eBioscience. ApoE-neutralizing (clone: HJ6.3) antibody was a gift from D. Holtzman^36^. We obtained DAPI (D3571) from Life Technologies, and mouse ITGB2 recombinant protein (LS-G14036-10) was from LSBio. We obtained mouse ApoE recombinant protein (MBS955382) from MyBioSource as well as CCR7 neutralizing (clone: 4B12) antibody (MAB3477)^78^ and VCAM1 antibody (BBA5) from R&D Systems. We obtained recombinant mouse CD44 protein (53953-M08H) from Sino Biological, MeX04 (4920) from Tocris Bioscience and Iba1 antibody (019-19741) from Wako.

### IL-33, neutralizing antibody and tamoxifen treatments in mice

For IL-33 treatment, we administered 1 ng recombinant murine IL-33 (in 2 μl sterile phosphate-buffered saline [PBS]) by intracerebroventricular injection at 0.3 μl min^−1^ at the following coordinates relative to the bregma: anteroposterior, −0.3 mm; mediolateral, +1.2 mm; dorsoventral, −2.3 mm. We euthanized the mice at different time points after injection as indicated in Results and figure legends.

Three to four hours before intracerebroventricular injection of IL-33, we administered 6 μg well-characterized neutralizing antibodies against ApoE, CCR7, ICAM1 and VCAM1 (in 3 μl) by intracerebroventricular injection at 0.3 μl min^−1^ at the following coordinates relative to the bregma: anteroposterior, −0.3 mm; mediolateral, +1.2 mm; dorsoventral, −2.3 mm.

To minimize the technical artifacts introduced by intracerebroventricular surgery on microglial activation, we used only the half-forebrain regions contralateral to the site of intracerebroventricular injection for our downstream analyses including our bulk/single-cell transcriptomic analyses, immunofluorescence staining and *in situ* hybridization experiments. We did not observe any notable microglial activation (that is, VCAM1 or MHC-II induction) introduced by the surgical procedures.

For tamoxifen administration, we dissolved tamoxifen powder (Sigma‐Aldrich) in corn oil at 20 mg ml^−1^ by shaking it overnight at 37 °C; we stored the working tamoxifen solution in the dark at 4 °C. Before administration, we incubated the tamoxifen solution in a 37 °C water bath for 5 min. To induce the nuclear translocation of CreERT2 and conditional knockout of the candidate gene, we intraperitoneally injected the tamoxifen solution (100 µl daily) for 4 consecutive days. We subsequently confirmed the knockout efficiency of *Vcam1* in microglia by real-time PCR.

### Flow cytometry and fluorescence-activated cell sorting

We performed flow cytometry and fluorescence-activated cell sorting as previously described^79^. In brief, we deeply anesthetized adult mice using isoflurane and then perfused them with ice-cold PBS. We minced the forebrain tissue into small pieces and mechanically dissociated them with a Dounce homogenizer on ice. We used a Percoll gradient (30%; Sigma-Aldrich) to remove myelin. We blocked the resultant mononuclear cell suspensions with an FcR blocker on ice for 10 min and then incubated them with antibody (all 1:100 dilution) in the dark on ice for 30 min. Next, we performed flow cytometry analysis and cell sorting with a BD Influx Cell Sorter. We used unstained controls to identify cell populations and visualized clear subpopulations of living microglia on scatter plots; the purity of microglial isolation was routinely > 90% according to reanalysis of the sorted cells. We analyzed the data using FlowJo software (version 10.8.2; Tree Star).

### Bulk RNA-seq library preparation

We sorted approximately 10,000 living microglia directly into RA1 Lysis Buffer and stored them at −80 °C until RNA extraction. We then extracted RNA from the microglia using a NucleoSpin RNA XS Kit (Macherey-Nagel) according to the manufacturer’s instructions. We immediately converted the extracted RNA into full-length complementary DNA (cDNA) using a SMART-Seq v4 kit (Takara Bio). Next, we quantified the concentrations of the cDNA libraries with Qubit (Thermo Fisher Scientific) and fragment lengths with Fragment Analyzer (Advanced Analytical). For library construction, we tagmented 1 ng cDNA by incubation with Tn5 transposase (Illumina) for 30 min at 55 °C and then amplified the tagmented cDNA by PCR for 12 cycles. We size-selected the libraries (100–600 bp) with AMPure XP beads (Beckman Coulter). We quantified the concentrations of the final libraries with Qubit (Thermo Fisher Scientific) and fragment lengths with Fragment Analyzer (Advanced Analytical). We performed paired-end sequencing using a NextSeq 500 or NovaSeq 6000 instrument (Novogene) according to the manufacturer’s instructions.

### Bulk RNA-seq analysis

We aligned the sequencing data to the mm10 mouse reference genome using STAR (version 2.7.0)^80^ and quantified the data using the *Rsubread* (version 2.4.3) package^81^ in R. We also performed differential expression analysis using the *DESeq2* (version 1.30.1) package^82^ in R. We defined differentially regulated genes (DEGs) as those with an adjusted *P* value < 0.05. We visualized the gene expression of DEGs under different conditions using Morpheus online software. Finally, we functionally annotated the DEGs by GO enrichment analysis and the STRING database.

### scRNA-seq library preparation

We generated scRNA-seq libraries using a Chromium Next GEM Single Cell 3′ Library Kit (v3.1 Chemistry; 10x Genomics) according to the manufacturer’s instructions. In brief, we counted sorted living CD11b^+^ cells on a hemocytometer. We mixed single-cell suspensions (400 cells µl^−1^) with reverse transcription reagent mix and loaded them into the chip for single-cell encapsulation. We immediately incubated the encapsulated cells in a thermocycler for reverse transcription. We obtained barcoded cDNA and used it for library construction according to the manufacturer’s instructions. We quantified the concentrations of the final libraries with Qubit (Thermo Fisher Scientific) and fragment lengths with Fragment Analyzer (Advanced Analytical). We performed paired-end sequencing of the libraries on a NovaSeq 6000 instrument (Novogene) according to the manufacturer’s instructions.

### scRNA-seq analysis

We performed scRNA-seq analysis as previously described^23,66^. For better identification of IL-33RM, we included a APP/PS1-PBS control sample from our previous study^23^. Demultiplexed FASTQ files (from Novogene) were aligned to mm10 mouse reference genome by Cell Ranger (v7.0.0) with the default settings^83^. Then, we performed downstream quality control (QC) and analyses using Seurat (v4.1.0). We further removed microglia with ≤500 genes, ≥15,000 unique molecular identifiers and ≥5% mitochondrial genes as second round of QC. After the QC, 72,519 microglia (from 86,288 CD11b^+^ cells) were retained for the downstream analysis.

We first performed samples integration to combine samples across conditions. We first performed the log-normalization on the matrices and identified highly variable features using the *FindVariableFeatures* function (*selection.method = vst* and *nfeatures* = *2000*). Then, we identified the anchoring features by the *FindIntegrationAnchors* function (*dims* = *1:20*) and used the identified anchors to combine samples across conditions using the *IntegrateData* function (*dims* = *1:20*). After scaling and linear dimensional reduction by *RunPCA* function (*npcs* = *50*), we use the top 30 principal components for graph-based clustering.

Graph-based clustering is performed by first using the *FindClusters* function (*resolution* = *0.3*) and followed by UMAP clustering using the *RunUMAP* function (*dims* = *1:30*). Microglial clustering across conditions is visualized by *Dimplot* function.

Differentially expressed genes across clusters/conditions is calculated using Wilcoxon rank-sum test in *FindAllMarkers* function (*logfc.threshold* = *0*). Adjusted *P* value < 0.05 is reconsidered as statistical significance.

Monocle3 (ref. ^84^) is used for pseudotemporal ordering analysis, which reconstructs the developmental trajectory of IL-33RM. We visualized the smoothed gene expression level (moving average of 1,000 microglia) with Morpheus.

### In situ hybridization

We performed in situ hybridization on formalin-fixed, paraffin-embedded mouse brain sections using an RNAscope Multiplex Fluorescent Reagent Kit v2 (323100) according to the manufacturer’s instructions. In brief, we deeply anesthetized the mice using isoflurane and perfused them with 20 ml 4% paraformaldehyde (PFA) in PBS; we isolated the half-brain contralateral to the injection site and fixed it in 4% PFA overnight. After fixation, we dehydrated, cleared, and infiltrated the brains with paraffin in a Revos Tissue Processor using a standardized processing protocol. We sectioned the embedded brain blocks at 6 μm using a microtome and stored them at 4 °C. Before in situ hybridization, we dried the brain sections for 1 h at 60 °C. Next, we deparaffinized the sections twice with Clear-Rite 3 for 5 min each time and then washed them twice with 100% ethanol for 2 min each time. After drying the sections at room temperature, we added hydrogen peroxide for 15 min to block endogenous peroxidase activity. We then washed the sections with DEPC-DPBS, submerged them in the provided target retrieval buffer, and boiled them for 15 min. Next, we applied protease plus to the sections for 15 min, washed the sections with DEPC-DPBS, and proceeded with the standardized probe hybridization procedure according to the manufacturer’s instructions. We purchased RNAscope probes targeting *Cx3cr1* and *Vcam1* from Advanced Cell Diagnostics. For Aβ plaques co-staining, we incubated the sections with an AF647-conjugated Aβ (6E10) antibody (1:1,000 dilution) overnight after in situ hybridization. We acquired confocal images using a Leica TCS SP8 confocal microscope. To examine the relative distance between the chemotactic microglia and Aβ plaques, we selected only Aβ plaques of a similar size for image acquisition and quantification; this minimizes the variations in microglia–Aβ interactions caused by Aβ plaque size and composition (that is, Aβ species and ApoE).

We manually quantified the proportions of *Vcam1*^+^ microglia and their relative distances to the nearest Aβ plaque using Leica Application Suite (LAS X) software (Leica) in a double-blinded manner. We considered microglia *Vcam1*^+^ when at least 2 *Vcam1* puncta were present. Given that Aβ plaques vary in size within the cortical regions, we measured the relative distance of microglia to the nearest Aβ plaque as the shortest distance between the center of the nucleus of each *Vcam1*^+^ microglia and the periphery of the Aβ plaque; this measurement minimizes the variation caused by differences in Aβ plaque size.

### Cell culture and in vitro wound-healing assay

The mouse BV2 microglial cell line was a generous gift from Dr. Douglas Golenbock’s laboratory, and the culture was performed as previously described^85^. In brief, BV2 cells were cultured in DMEM (Gibco) supplemented with 10% heat inactivated fetal and penicillin/streptomycin, and maintained in 100 mm plates in a humidified incubator containing 5% CO_2_ at 37 °C. For subculturing, cells were dissociated by 0.25% trypsin in PBS incubated at 37 °C for 2 min, transferred into 50 ml falcon, centrifugated at 800 x *g* for 5 min, and then resuspend in culture medium. Cell replating was performed once the confluency of the culture dish reached 80%-90%, and 1:20 dilution ratio was applied for cell passaging in every 3 or 4 days. Experiments were performed only when cells have been passaged for at least twice after thawing, and all cells were discarded once they reached P15.

To examine the migratory capacity of BV2 microglial cells, we performed wound-healing assay in Ibidi Culture-Insert 2 well 24 (80242, Ibidi) following manufacturer’s instruction. Briefly, 75,000 BV2 cells were seeded into chambers on both sides of the insert and cultured for 18 h. The inserts were then removed from the wells, and cells were washed by fresh medium, followed by treatment of VCAM1/ICAM1/CCR7 neutralizing antibody and IL-33. Immediately after treatment starts, the culture plate was placed into a ZEISS Celldiscoverer 7 (CD7) for automated live cell imaging.

In CD7, two views were selected along the gap between seeded cells and imaged for 24 h with 0.5-h interval. Images were analyzed using ImageJ (version 1.53) to measure the area covered by migrating cells. The area differences between each time point and starting time were calculated and averaged from two views in the same well as follows:

$${\mathrm {cell}}\,{\mathrm {covered}}\,{\mathrm {area}}\,\left( \% \right)=\frac{{A}_{t}-{A}_{t0}}{{A}_{t0}}\times 100 \%,$$

where *A*_*t*_ is the gap area covered by cell measured at *t* hour, and *A*_*t0*_ is the gap area covered by cell at starting time point. Student’s *t*-test is used for statistical comparison between groups.

### Immunofluorescence and immunohistochemical staining of human formalin-fixed, paraffin-embedded sections

We first deparaffinized and rehydrated the sections with Richard-Allan Scientific Signature Series Clear-Rite 3 (Thermo Scientific) and graded ethanol solutions. To identify VCAM1^+^ microglia in the brains of patients with AD, we treated the sections with sodium citrate buffer (10 mM sodium citrate, pH 6.0) for 20 min using a pressure cooker for epitope retrieval. To reduce background autofluorescence, we pre-treated the sections using the protocol adapted from Sun et al with slight modifications^86^. Then, we incubated the sections with 0.2% thioflavin S (Sigma-Aldrich) in 50% ethanol for 8 min followed by treatment with concentrated PBS in the dark for 10 min at 4 °C. After thioflavin S labeling, we blocked the sections with 10% goat serum for 1 h at room temperature and then incubated them with an anti-VCAM1 antibody (1:50 dilution) and anti-Iba1 antibody (1:200 dilution) in a dark, humid chamber overnight at 4 °C. After washing, we incubated the sections with secondary antibodies (all diluted 1:500) in the dark for 1 h at room temperature followed by SYTOX Green Nucleic Acid Stain (Thermo Fisher Scientific; 1:120,000 dilution) in the dark for 5 min. We subsequently mounted the sections using ProLong Diamond Antifade Mountant (Thermo Fisher Scientific) and stored them in the dark at 4 °C. We collected the images using a Zeiss LSM 980 microscope with Airyscan 2, and processed and analyzed them manually with ZEN software (version 3.3; Zeiss).

We used double immunohistochemical staining to examine the microglial interaction with Aβ on AD patient brains, as previously described^74^. In brief, we treated the deparaffinized, rehydrated sections with sodium citrate buffer (10 mM sodium citrate, pH 6.0) for 25 min, and followed by blocking and quenching of the peroxidase activities. We then incubated the sections with anti-Aβ antibody (clone: NAB228) (1:500 dilution) and anti-Iba1 antibody (1:100 dilution) overnight at 4 °C. Next, the sections were washed and incubated with horseradish peroxidase-labeled anti-mouse Ig and alkaline phosphatase (AP)-labeled anti-rabbit Ig (HK597-50K, Double Staining Kit, BioGenex). We developed the sections with 3,3′-diaminobenzidine (DAB) and fast red substrate (BioGenex), and further counterstained before mounting. Images acquisition is performed with a Zeiss Axio Scan.Z1 scanner and analyzed with ZEN software (version 3.3; Zeiss). To analyze the microglial Aβ interaction, we selected 20 fields per section and processed with *Colour Deconvolution* function in Fiji software (ImageJ v1.53c) to separate the images into three different channels: DAB, Fast Red, and hematoxylin. After adjusting the thresholds, we determined the total Aβ area and Iba-stained Aβ area by the *Create Selection* function and followed by *Analyze* function. Aβ plaque–microglial interaction (% total Aβ) area is calculated by dividing Iba-1–stained Aβ area by the total Aβ area.

To analyze Aβ deposition in the AD brain, we performed antigen retrieval by formic acid for 5 min at room temperature. Followed by quenched with 3% hydrogen peroxide, we stained the sections with a mouse anti-Aβ antibody (clone: NAB228) (1:500 dilution) overnight at 4 °C. Next, we stained the sections with horseradish peroxidase-labeled anti-mouse IgG (SS Polymer) and developed them with DAB substrate (BioGenex). 10 images were selected from each section for downstream analysis. We first performed background subtraction and threshold adjustment, then we used the *Analyze Particles* function to determine the total Aβ area for each section. Amyloid plaque load (% area) = total Aβ area / total image area (100 mm^2^).

For the imaging analyses, two independent researchers performed section staining, image acquisition, and image quantification. Images were quantified in a blinded manner.

### Immunofluorescence staining of mouse brain sections

We deeply anesthetized adult mice using isoflurane and perfused them with ice-cold 4% PFA. We isolated their brains and fixed them overnight in 4% PFA. We then prepared 50-μm floating sections using a vibratome.

To examine the microglial transition to an MHC-II^+^ phagocytic phenotype and Aβ clearance after IL-33 treatment, we performed antigen retrieval on floating sections using Tris-EDTA (pH 9.0) at 85 °C for 15 min. After washing, we blocked the sections with 1% BSA, 4% horse serum, and 0.4% Triton X-100 in PBS at room temperature for 30 min followed by incubation with anti-Iba1 antibody (1:1,000 dilution), MeX04 (1:30,000 dilution from 1 mg/mL stock), and anti-MHC-II antibody (1:1,000 dilution) overnight at 4 °C. After washing, we incubated the sections with the goat anti-rabbit IgG (H + L) AF647 antibody and goat anti-rat IgG (H + L) AF488 antibody (Invitrogen; 1:1,000 dilution) overnight at 4 °C. We acquired confocal images using a Zeiss LSM880 confocal microscope. To examine microglia–Aβ interactions, we selected only Aβ plaques of a similar size for image acquisition and quantification; this minimizes the variations in microglia–Aβ interactions caused by Aβ plaque size and composition (that is, Aβ species and ApoE).

For each sample, we stained two or three brain sections (∼150–200 μm apart, near the hippocampus). To quantify the proportions of Aβ plaque-associated microglia and MHC-II^+^ microglia, we analyzed 4 areas per section. For each sample, we obtained and analyzed at least 200 microglia using ImageJ (version 1.53). To quantify the areas of Aβ plaques, we tile-scanned and analyzed sections from the entire cortex using the *Analyze Particles* function in ImageJ (version 1.53).

To study the microglial chemotactic response to chemoattractant-coated beads, we performed antigen retrieval on floating sections using Tris-EDTA (pH 9.0) at 85 °C for 15 min. After washing, we blocked the sections with 1% BSA, 4% horse serum, and 0.4% Triton X-100 in PBS at room temperature for 30 min followed by incubation with anti-Iba1 antibody (1:1,000 dilution), DAPI (1:5,000 dilution), and anti-MHC-II antibody (1:1,000 dilution) overnight at 4 °C. After washing, we incubated the sections with the goat anti-rabbit IgG (H + L) AF647 antibody and goat anti-rat IgG (H + L) AF488 antibody (Invitrogen; 1:1,000 dilution) overnight at 4 °C. We acquired confocal images using a Zeiss LSM880 confocal microscope. During image acquisition, we first located and imaged the protein-coated beads under the brightfield setting and subsequently switched to the confocal setting for the fluorescence imaging of microglia.

For each sample, we stained and analyzed three consecutive brain sections (∼150 μm covering the entire volume of the injected beads). We then analyzed and manually calculated the average numbers of microglia and MHC-II^+^ microglia surrounding and within the bead area by using ImageJ (version 1.53). We quantified the bead area as the darkened area in the brightfield images (Extended Data Fig. 4a,b).

### Analysis of microglial chemotaxis toward chemoattractant-coated beads

To examine microglial chemotaxis toward a given chemoattractant in vivo, we followed a stereotactic injection method modifications to the injection material and site^47^. To generate chemoattractant-coated beads, we first washed anti-His tag beads twice with 0.1% BSA in PBS. Then, we incubated 5 μl beads with 30 ng recombinant ApoE, CD44, or ITGB2 protein for 30 min at 4 °C. After two rounds of washing with 0.1% BSA in PBS, we resuspended the beads in 30 μl 1% BSA in PBS to achieve a concentration of 1 ng µl^−1^ chemoattractant-coated beads and immediately proceeded with stereotactic injection. One to two hours before intracerebroventricular injection of IL-33, we injected the chemoattractant-coated beads into the mice at 0.3 μl min^−1^ at the following coordinates relative to the bregma: anteroposterior, 0 mm; mediolateral, ±2.0 mm; and dorsoventral, −1.5 mm. we euthanized the mice 24 h after intracerebroventricular injection of IL-33.

We performed lipidation of recombinant ApoE as previously described^60^. In brief, we prepared reconstituted ApoE particles by a cholate dialysis method using a ApoE:POPC:cholesterol molar ratio of 1:50:10. We analyzed the reconstituted ApoE particles by nondenaturing gradient PAGE.

For co-injection of ApoE beads and rVCAM1 protein, we prepared ApoE beads as mentioned above and resuspended them in 30 μl 3 ng μl^−1^ VCAM1 or Fc-control in PBS.

### Measurement of plasma and cerebrospinal fluid VCAM1 levels and levels of AD-related biomarkers

We measured the Aβ_42/40_ ratio as well as tau, p-tau181 (tau phosphorylated at threonine-181), and neurofilament light polypeptide levels in 350 μL plasma with a Quanterix Accelerator Laboratory using a Quanterix Simoa NF-light Assay Advantage Kit (103186), Neurology 3-Plex A Kit (101995), and pTau-181 Advantage V2 Kit (103714) as appropriate. We analyzed VCAM1 levels in plasma and CSF using a Human VCAM1-/CD106 Quantikine ELISA Kit (DVC00) according to the manufacturer’s instructions.

### Statistics and reproducibility

All statistical methods were reported in the figures, figure legends, and methods. All RNA-seq experiments, that is, both bulk and single-cell, were repeated for two batches with similar results. Analysis was performed using data combined from two batches. All other experiments were repeated for three to five batches with similar results.

No statistical methods were used to predetermine sample sizes but sample sizes primarily based on the common standards and practices of similar types of experiments in the field: *n* = 4–5 mice for bulk RNA-seq (ENCODE: https://www.encodeproject.org/data-standards/rna-seq/long-rnas/) and in situ hybridization experiments^9,67,87^ as well as *n* = 6–13 mice for microglia staining and AD pathology measurement^31,32,35,50,88^.

#### Randomization

No randomization method was used to allocate animals to experimental groups. For human staining and ELISA measurement, patient samples were selected based on availability and quality. For plasma ELISA, we selected 32 samples (M = 7, F = 25; NC = 15, AD = 17; age = 67–87 years; MoCA = 4–30) from our Chinese cohort.

#### Blinding

All analyses, except bioinformatic analysis of sequencing, were performed in a double-blinded manner. Bulk RNA-seq and single-cell RNA-seq analyses were performed without bias because experimental conditions are required for result interpretation and downstream analysis, such as pseudotime trajectory projection. However, sequencing results were validated by independent approaches, including in situ hybridization and immunofluorescent staining, in a double-blinded manner. For human sample analysis, investigators were blinded to allocation during experiments and outcome assessment for imaging, and ELISA analysis. For immunofluorescent imaging analysis, data collection and analysis were performed in a double-blinded manner.

#### Data exclusion

For single-cell transcriptomic analysis, microglia with < 200 genes, > 20,000 unique molecular identifiers and > 20% mitochondrial genes were excluded. These parameters are commonly adopted as quality-check for single-cell RNA-seq data. No sample was excluded in animal and cell culture experiments. In Fig. 6b,c, datapoints were excluded due to undetectable level of pTau181 or NfL. In Fig. 6e and Extended Data Fig. 6d, datapoints with 1) CSF VCAM1 level below detection level, 2) incomplete *APOE4* genotype information and 3) poor immunohistochemical staining of microglia and Aβ were excluded from the analysis. For other experiments, no datapoint was excluded from the analysis.

For bulk RNA-seq, we performed differential expression analysis using the *DESeq2* package in R. We considered genes to be differentially regulated if the adjusted *P* value was < 0.05. For scRNA-seq, we performed differential analysis with the Wilcoxon rank-sum test using the *FindAllMarkers* function with the parameter *logfc.threshold* = *0*. We set the level of statistical significance to an adjusted *P* value < 0.05. We performed all other statistical analyses using GraphPad Prism 9.

### Reporting summary

Further information on research design is available in the Nature Portfolio Reporting Summary linked to this article.

### Supplementary information

Reporting Summary

Supplementary Table Demographic characteristics of human cohorts

### Source data

Source Data Fig. 1 Statistical source data.

Source Data Fig. 2 Statistical source data.

Source Data Fig. 3 Statistical source data.

Source Data Fig. 4 Statistical source data.

Source Data Fig. 5 Statistical source data.

Source Data Fig. 6 Statistical source data.

Source Data Extended Data Fig. 1 Statistical source data.

Source Data Extended Data Fig. 2 Statistical source data.

Source Data Extended Data Fig. 3 Statistical source data.

Source Data Extended Data Fig. 4 Statistical source data.

Source Data Extended Data Fig. 5 Statistical source data.

Source Data Extended Data Fig. 6 Statistical source data.

## Data Availability

All raw sequencing data and processed data are available at Gene Expression Omnibus repository under accession number GSE208006. All other data supporting the findings of this study are available as source data files or from the corresponding author, N.Y.I. (boip@ust.hk), upon reasonable request.

## Code availability

All code used for bulk RNA-seq and scRNA-seq analysis can be found at https://github.com/sflau123/Vcam1-ApoE_microglia_AD.git. Others are available from the corresponding author, N.Y.I. (boip@ust.hk), upon request.
