# Supplementary material for: The VCAM1–ApoE pathway directs microglial chemotaxis and alleviates Alzheimer’s disease pathology
Source: Nat Aging. 2023 Sep 21;3(10):1219–36. doi: 10.1038/s43587-023-00491-1 (PMC10570140; doi:10.1038/s43587-023-00491-1)
Supplement: Supplementary file 1 — Reporting Summary [file 43587_2023_491_MOESM1_ESM.pdf]

nature portfolio

Corresponding author(s): Nancy Y Ip

Last updated by author(s): Jul 31, 2023

Reporting Summary

Nature Portfolio wishes to improve the reproducibility of the work that we publish. This form provides structure for consistency and transparency in reporting. For further information on Nature Portfolio policies, see our Editorial Policies and the Editorial Policy Checklist.

Statistics

For all statistical analyses, confirm that the following items are present in the figure legend, table legend, main text, or Methods section.

n/a	Confirmed	
☐	☒	The exact sample size (*n*) for each experimental group/condition, given as a discrete number and unit of measurement
☐	☒	A statement on whether measurements were taken from distinct samples or whether the same sample was measured repeatedly
☐	☒	The statistical test(s) used AND whether they are one- or two-sided<br>*Only common tests should be described solely by name; describe more complex techniques in the Methods section.*
☒	☐	A description of all covariates tested
☐	☒	A description of any assumptions or corrections, such as tests of normality and adjustment for multiple comparisons
☐	☒	A full description of the statistical parameters including central tendency (e.g. means) or other basic estimates (e.g. regression coefficient) AND variation (e.g. standard deviation) or associated estimates of uncertainty (e.g. confidence intervals)
☐	☒	For null hypothesis testing, the test statistic (e.g. *F*, *t*, *r*) with confidence intervals, effect sizes, degrees of freedom and *P* value noted<br>*Give P values as exact values whenever suitable.*
☒	☐	For Bayesian analysis, information on the choice of priors and Markov chain Monte Carlo settings
☒	☐	For hierarchical and complex designs, identification of the appropriate level for tests and full reporting of outcomes
☒	☐	Estimates of effect sizes (e.g. Cohen's *d*, Pearson's *r*), indicating how they were calculated

Our web collection on statistics for biologists contains articles on many of the points above.

Software and code

Policy information about availability of computer code

Data collection	Flow cytometry data were acquired using BD Influx Cell Sorter.<br>For in situ hybridization staining, confocal images were acquired using a Leica TCS SP8 confocal microscope.<br>For mice immunofluorescence staining, confocal images were acquired using a Zeiss LSM880 confocal microscope with Airyscan.<br>For human immunofluorescence staining, confocal images were acquired using a Zeiss LSM980 microscope with Airyscan 2.<br>For human immunohistochemical staining, images were acquired using Zeiss Axio Scan.Z1 scanner.<br>Please refer to the Methods section for details.
Data analysis	Flow cytometry analysis was performed using FlowJo v10.8.2.<br>Bulk RNA-seq analysis was performed using STAR v2.7.0, Rsubread v2.4.3 package and DESeq2 v1.30.1 package in R v4.0.3.<br>Single-cell RNA-seq analysis was performed using Cellranger v7.0.0, Seurat v4.1.0, and Monocle3.<br>ZEN (version 3.3); Fiji software (ImageJ v1.53c); ImageJ (version 1.53) were used to quantify all imaging data from immunofluorescence and immunohistochemical staining.<br>Leica Application Suite (LAS X) software (Leica) was used to quantify all imaging data from in situ hybridization staining.<br>Graph Pad (Prism v9.0) was used to perform statistical analyses presented and generated plots throughout the manuscript.<br>Please refer to the Methods section for details.

For manuscripts utilizing custom algorithms or software that are central to the research but not yet described in published literature, software must be made available to editors and reviewers. We strongly encourage code deposition in a community repository (e.g. GitHub). See the Nature Portfolio guidelines for submitting code & software for further information.

Data

Policy information about availability of data

All manuscripts must include a data availability statement. This statement should provide the following information, where applicable:

- Accession codes, unique identifiers, or web links for publicly available datasets
- A description of any restrictions on data availability
- For clinical datasets or third party data, please ensure that the statement adheres to our policy

All raw sequencing data and processed data are available at Gene Expression Omnibus repository with accession: GSE208006. Other data are available from the corresponding author, Dr. Nancy Y. Ip (boip@ust.hk), upon request.

Research involving human participants, their data, or biological material

Policy information about studies with human participants or human data. See also policy information about sex, gender (identity/presentation), and sexual orientation and race, ethnicity and racism.

Reporting on sex and gender	Human samples, including plasma, CSF, and FFPE brain sections, were obtained from both sexes. We did not observe obvious sex-specific effect in our analysis, as indicated in the main figures and extended figures.
Reporting on race, ethnicity, or other socially relevant groupings	Plasma ELISA analysis is performed using a Chinese cohort data, we previously collected and published (37). In brief, the cohort consists of 345 patients with AD and 345 health controls (all aged ≥60 years). All individuals underwent medical history assessment, clinical assessment, cognitive and functional assessments using the Montreal Cognitive Assessment test, and neuroimaging assessment by MRI. We randomly selected 32 samples for our ELISA analysis.<br><br>CSF ELISA and immunohistochemical staining is performed using AD samples from the South West Dementia Brain Bank (SWDBB), which receives approval from North Somerset and South Bristol Research Ethics Committee to operate as a research tissue bank.
Population characteristics	For plasma ELISA analysis, we randomly selected 32 samples (NC = 15, AD = 17;M = 7, F = 25; age = 67–87; MoCA = 4–30) for our Chinese cohort for analysis.<br>For CSF ELISA and immunohistochemical analysis, we selected 35 AD samples (M = 18, F = 17; age = 54-96). The detailed population characteristics of the SWDBB samples, including age, sex, APOE4 genotype, CSF VCAM1 level, and microglia–Aβ interaction are shown in Extended Data Table 1.
Recruitment	We collected plasma samples from healthy controls of Hong Kong Chinese descent and patients with AD aged ≥60 years who visited the Specialist Outpatient Department of the Prince of Wales Hospital at the Chinese University of Hong Kong from April 2013 to February 2018. The clinical diagnosis of AD was based on the criteria for AD in the Diagnostic and Statistical Manual of Mental Disorders, Fifth Edition (DSM-5). All participants underwent medical history assessment, clinical assessment, cognitive and functional assessments using the Montreal Cognitive Assessment, and neuroimaging by magnetic resonance imaging. We excluded participants with any neurological disease other than AD or any psychiatric disorder. We recorded participants' age, sex, years of education, medical history, history of cardiovascular disease (i.e., heart disease, hypertension, diabetes mellitus, and hyperlipidemia), and white blood cell counts.<br><br>For postmortem formalin-fixed, paraffin-embedded brain sections and cerebrospinal fluid (CSF) samples, the clinical diagnosis of AD was based on the DSM-5 criteria for AD. For our initial sample selection from the SWDBB, we excluded subjects with neurodegenerative diseases other than AD, vascular diseases, an intoxicated state or infection at the time of death, prions, inflammatory diseases, structural brain disorders, metabolic/nutritional diseases, trauma, delirium, genetic disorders (e.g., Down syndrome), or systemic diseases other than AD. Population characteristics, CSF VCAM1 level and microglia–Aβ are shown in Extended Data Table 1.
Ethics oversight	The study was approved by the Clinical Research & Ethics Committees of Joint Chinese University of Hong Kong-New Territories East cluster for Prince of Wales Hospital (CREC Ref no. 2015.461), Kowloon Central Cluster/Kowloon East Cluster for Queen Elizabeth Hospital (KC/KE-15-0024/FR-3), and Human Participants Research Panel of the Hong Kong University of Science and Technology (CRP#180 and CRP#225). All participants provided written informed consent for both study participation and sample collection.<br><br>We obtained postmortem formalin-fixed, paraffin-embedded brain sections and cerebrospinal fluid (CSF) samples from patients with AD from the South West Dementia Brain Bank (SWDBB), which receives approval from North Somerset and South Bristol Research Ethics Committee to operate as a research tissue bank (REC reference number: 23/SW/0023).

Note that full information on the approval of the study protocol must also be provided in the manuscript.

Field-specific reporting

Please select the one below that is the best fit for your research. If you are not sure, read the appropriate sections before making your selection.

☒ Life sciences ☐ Behavioural & social sciences ☐ Ecological, evolutionary & environmental sciences

For a reference copy of the document with all sections, see nature.com/documents/nr-reporting-summary-flat.pdf

Life sciences study design

All studies must disclose on these points even when the disclosure is negative.

Sample size	No power calculation was performed prior to study design, sample size of all animal experiments was determined based on previous experience. We randomized mice into experimental groups and chose sample sizes primarily based on the common standards and practices of similar types of experiments in the field: n = 4–5 mice for bulk RNA sequencing (RNA-seq) (ENCODE: https://www.encodeproject.org/data-standards/rna-seq/long-rnas/) and in situ hybridization experiments (9,67,73) as well as n = 6–13 mice for microglia staining and Alzheimer's disease (AD) pathology measurement (31,32,35,50,74). Samples size of human samples was determine based on sample availability. All number of mice and human samples were indicated in the manuscript.
Data exclusions	For single-cell transcriptomic analysis, microglia with < 200 genes, > 20,000 unique molecular identifiers, and >20% mitochondrial genes were exlcuded. These parameters are commonly adopted as quality-check for single-cell RNA-seq data.<br>No sample was excluded in animal and cell culture experiments.<br>For human samples, we only selected samples with postmortem delay < 24 hours for CSF ELISA measurement. This minimizes plasma contamination due to BBB breakdown. Furthermore, AD patients with poor quality of FFPE sections were excluded from the analysis.
Replication	Bulk RNA-seq library were constructed in 2 batches (n = 1-2 per batch). All experiments were repeated for at least 3 batches and all attempts at replication were successful.
Randomization	All animal and cell culture experiments were randomly allocated into experimental conditions. For human staining and ELISA measurement, patient samples were selected based on availability and quality.
Blinding	All analyses, except bioinformatic analysis of sequencing, were performed in a double-blinded manner. Bulk RNA-seq and single-cell RNA-seq analyses were performed without bias because experimental conditions are required for result interpretation and downstream analysis, such as pseudotime trajectory projection. However, sequencing results were validated by independent approaches, including in situ hybridization and immunofluorescent staining, in a double-blinded manner.

Reporting for specific materials, systems and methods

We require information from authors about some types of materials, experimental systems and methods used in many studies. Here, indicate whether each material, system or method listed is relevant to your study. If you are not sure if a list item applies to your research, read the appropriate section before selecting a response.

Materials & experimental systems

n/a	Involved in the study	
☐	☒	Antibodies
☐	☒	Eukaryotic cell lines
☒	☐	Palaeontology and archaeology
☐	☒	Animals and other organisms
☒	☐	Clinical data
☒	☐	Dual use research of concern
☒	☐	Plants

Methods

n/a	Involved in the study	
☒	☐	ChIP-seq
☐	☒	Flow cytometry
☒	☐	MRI-based neuroimaging

Antibodies

Antibodies used	Primary antibodies:<br>We obtained AF647-conjugated Aβ (clone: 6E10) antibody (803021), APC-conjugated MHC-II (clone: M5/114.15.2) antibody (107614), FITC-conjugated VCAM1 (clone: MVCAM.A) antibody (105706), and MHC-II (I-A/I-E) (clone: M5/114.15.2) antibody (107601) from BioLegend. We obtained ICAM1-neutralizing (clone: YN1/1.7.4) antibody (BE0020) (76,77) and VCAM1-neutralizing (clone: M/K-2.7) antibody (BE0027) (69,78) from Bio X Cell. We obtained AF488-conjugated CD11b (clone: M1/70) antibody (53-0112-82), APC-conjugated CD11b (clone: M1/70) antibody (17-0112-83), and biotinylated CD11b (clone: M1/70) antibody (13-0112-82) from eBioscience. ApoE-neutralizing (clone: HJ6.3) antibody was a gift from Dr. David Holtzman (36).We obtained CCR7-neutralizing (clone: 4B12) antibody (MAB3477) (79) and VCAM1 antibody (BBA5) from R&D Systems. We obtained Iba1 antibody (019-19741) from Wako.<br><br>Secondary antibodies:<br>Horseradish peroxidase (HRP)-labeled anti-mouse Ig and alkaline phosphatase (AP)-labeled anti-rabbit Ig (Double Staining Kit, BioGenex).<br>HRP-labeled anti-mouse IgG (SS Polymer)<br>Goat anti-rabbit IgG (H+L) Alexa Fluor(AF) 647 antibody (Invitrogen)<br>Goat anti-rat IgG (H+L) AF488 antibody (Invitrogen)
Validation	All commercial available antibodies were well-characterized and verified by the company. These antibodies were the most commonly used for immunostaining, flow cytometry analysis in the field of neuroscience and immunology. All neutralizing antibodies was previously validated and published: ICAM1-neutralizing (clone: YN1/1.7.4) antibody (BE0020) (76,77); VCAM1-neutralizing (clone: M/

K-2.7) antibody (BE0027) (69,78); CCR7-neutralizing (clone: 4B12) antibody (MAB3477) (79), ApoE-neutralizing (clone: HJ6.3) antibody (36).

Eukaryotic cell lines

Policy information about cell lines and Sex and Gender in Research

Cell line source(s)	BV2 microglial cell line was a generous gift from Dr. Douglas Golenbock's laboratory, and the culture was performed as previously described (86).
Authentication	We performed RNA-sequencing analysis (data not shown) to confirm the identify of microglia, especially the expression of Cx3cr1, CD45, CD11b and negative for markers of other neural cells.
Mycoplasma contamination	The BV2 cells were tested negative for mycoplasma contamination.
Commonly misidentified lines (See ICLAC register)	No commonly misidentified cell lines were used.

Animals and other research organisms

Policy information about studies involving animals; ARRIVE guidelines recommended for reporting animal research, and Sex and Gender in Research

Laboratory animals	We obtained four mouse strains from the Jackson Laboratory: APP/PS1 transgenic mice (B6C3-Tg[APPswe, PSEN1dE9]85Dbo), which were generated by incorporating a human/murine APP construct bearing the Swedish double mutation and exon-9–deleted PSEN1 mutation (APPswe + PSEN1/dE9); ApoE-knockout mice (B6.129P2-Apoetm1Unc/J); Cx3cr1creERT2 mice (B6.129P2[Cg]-Cx3cr1tm2.1[cre/ERT2]Litt) in which a microglia-specific promoter controls CreERT2 expression; and Vcam1loxP/loxP mice (B6.129[C3]-Vcam1tm2Flv/J), which have loxP sites on either side of the cytokine-responsive promoter region and exon 1 of the Vcam1 gene. ST2loxP/loxP mice were generated in this study, and available upon request. ST2-deficient mice were provided by Dr. Andrew N. J. McKenzie of the Medical Research Council Laboratory of Molecular Biology in Cambridge, UK (73). We confirmed the genotypes of the mice by PCR analysis of ear biopsy specimens.<br>We used mice of both sexes for the experiments and performed all experiments using groups of sex- and age-matched (10–11 months old) mice. All mice were housed at the Hong Kong University of Science and Technology (HKUST) Animal and Plant Care Facility. We housed mice of the same sex in temperature and humidity-controlled environment, on a 12-h light/dark cycle and provided them with food and water ad libitum.
Wild animals	No wild animals were used.
Reporting on sex	Mice of both sexes were used in the study. Consistent with previous findings (23,49,70–72), we did not observe an obvious sex-specific microglial response toward interleukin-33 (IL-33).
Field-collected samples	No field-collected samples were used.
Ethics oversight	We performed all animal experiments in accordance with protocols #A19054 and #V190021, which were approved by the Animal Care Committee of HKUST.

Note that full information on the approval of the study protocol must also be provided in the manuscript.

Flow Cytometry

Plots

Confirm that:

☒ The axis labels state the marker and fluorochrome used (e.g. CD4-FITC).

☒ The axis scales are clearly visible. Include numbers along axes only for bottom left plot of group (a 'group' is an analysis of identical markers).

☒ All plots are contour plots with outliers or pseudocolor plots.

☒ A numerical value for number of cells or percentage (with statistics) is provided.

Methodology

Sample preparation	We deeply anesthetized adult mice using isoflurane and then perfused them with ice-cold PBS. We isolated the forebrain, minced them into small pieces, then mechanically dissociated them with a Dounce homogenizer on ice. We used a Percoll gradient (30%; Sigma-Aldrich) to remove myelin. We blocked the resultant mononuclear cell suspensions with an FcR blocker for 10 min on ice, then incubated them with antibody in the dark for 30 min on ice.
Instrument	BD Influx Cell Sorter
Software	FlowJo software (Tree Star).

Cell population abundance	The purity of microglial isolation was routinely >90% according to a reanalysis of the sorted cells
Gating strategy	We used FMO unstained controls to identify cell populations and visualized clear subpopulations of living microglia on scatter plots

☒ Tick this box to confirm that a figure exemplifying the gating strategy is provided in the Supplementary Information.
